# Exploring the Therapeutic Potential of Antidiabetic Drugs in Cardiac Arrhythmia Management: A Drug Target Mendelian Randomization Study

**DOI:** 10.1002/joa3.70241

**Published:** 2025-12-04

**Authors:** Zheng‐Qi Song, Zhi‐Bo Zhou, Bo‐Xiang Wang, Sheng‐Ke Wu, Yi‐Han Sun, Yi‐He Chen, Su‐Yin Feng, Run‐Feng Sun

**Affiliations:** ^1^ Donghai County People's Hospital ‐ Jiangnan University Smart Healthcare Joint Laboratory Donghai County People's Hospital (Affiliated Kangda College of Nanjing Medical University) Lianyungang City Jiangsu Province China; ^2^ Cardio‐Cerebral Vascular Disease Prevention and Treatment Innovation Center Donghai County People's Hospital, (Affiliated Kangda College of Nanjing Medical University) Lianyungang City Jiangsu Province China; ^3^ The First Clinical Medical College Wenzhou Medical University Zhejiang China; ^4^ Second College of Clinical Medical Wenzhou Medical University Wenzhou China; ^5^ Department of Cardiology The First Affiliated Hospital of Wenzhou Medical University Zhejiang China

**Keywords:** antidiabetic drugs, cardiac arrhythmias, drug target, Mendelian randomization

## Abstract

**Background:**

Cardiac arrhythmias pose a major health concern while the role of antidiabetic medications in cardiac arrhythmic risks is not fully understood.

**Method:**

We conducted a two‐sample Mendelian Randomization (MR) analysis using genetic instruments extracted from hemoglobin A1C (HbA1c) as proxies for antidiabetic drug targets to evaluate their causal relationship with five cardiac arrhythmias derived from the Finngen database. Summary‐data‐based Mendelian randomization (SMR) utilizing gene expression data from the eQTLgen consortium was further employed to assess the role of antidiabetic drug targets in cardiac arrhythmias from the gene expression perspective.

**Results:**

Three significant associations were identified. Sulfonylurea targets KCNJ11/ABCC8 were associated with a decreased incidence of paroxysmal tachycardia (OR: 0.69, 95% CI: 0.56, 0.86, *P*_FDR_ = 0.022). Sodium‐glucose cotransporter 2 inhibitor (SGLT2i) target SLC5A2 was linked to a reduced risk of right bundle branch block (OR: 0.85, 95% CI: 0.77, 0.94, PFDR = 0.022), and thiazolidinediones (TZDs) targeting RXRB were associated with a lowered atrial fibrillation occurrence (OR: 0.88, 95% CI: 0.82, 0.94, *P*_FDR_ = 0.019). No significant relationships were found between any antidiabetic drug targets and left bundle branch block or atrioventricular block. SMR analysis indicated that lowered expression of KCNJ11 was related to a decreased paroxysmal tachycardia risk (OR: 1.05, 95% CI: 1.01, 1.08, *P*_SMR_ = 0.010), further confirming the role of KCNJ11 in paroxysmal tachycardia.

**Conclusion:**

Our findings suggest that several antidiabetic drug targets may have potential therapeutic applications in the management of cardiac arrhythmias.

## Graphical abstract

Mendelian randomization analyses suggest that genetic proxies for antidiabetic drug targets—KCNJ11/ABCC8, SLC5A2, and RXRB—reduce the risk of paroxysmal tachycardia (PT), right bundle branch block (RBBB), and atrial fibrillation (AF), respectively.

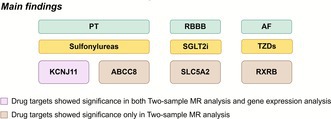

**Abbreviations**
AF: Atrial fibrillation
AVB: Atrioventricular block
DPP‐4i: Dipeptidyl peptidase‐4 inhibitor
eQTL: Expression quantitative trait loci
FDR: False discovery rate
GLP‐1RA: Glucagon‐like peptide‐1 receptor agonist
GWAS: Genome‐wide association study
HbA1c: Hemoglobin A1C
HEIDI: Heterogeneity in the dependent instrument
IV: Instrumental variable
IVW: Inverse variance weighted
K_ATP_
: ATP‐sensitive potassium
LBBB: Left bundle branch block
LD: Linkage disequilibrium
MR: Mendelian randomization
MR‐PRESSO: MR‐Pleiotropy RESidual Sum and Outlier
OR: Odds ratio
PPARG: Peroxisome proliferator‐activated receptor gamma
PT: Paroxysmal tachycardia
RBBB: Right bundle branch block
RXRB: Retinoid X Receptor β
SD: Standard deviation
SGLT2i: Sodium‐glucose cotransporter‐2 inhibitor
SMR: Summary‐data‐based Mendelian randomization
SNP: Single nucleotide polymorphism
STROBE‐MR: Strengthening the Reporting of Observational Studies in Epidemiologic Trials
T2DM: Type 2 diabetes mellitus
TZDs: Thiazolidinediones

## 1 Introduction

Cardiac arrhythmia is a leading cause of morbidity and mortality worldwide, accounting for approximately 50% of sudden cardiac deaths and posing a substantial healthcare burden [1]. Emerging evidence suggests that metabolic factors, particularly glucose homeostasis, play a critical role in arrhythmogenesis [2]. Glucose‐lowering drugs help regulate blood glucose levels and control the progression of diabetes, thereby reducing the incidence of arrhythmias [3]. However, the use of these medications may also cause fluctuations in blood glucose levels, which can trigger oxidative stress, activate the sympathoadrenal system, and disrupt electrolyte balance, all of which contribute to arrhythmia development [3, 4]. Furthermore, due to differences in their mechanisms of action, various glucose‐lowering drugs may have varying effects on cardiac rhythm, highlighting the need for a more detailed evaluation of their electrophysiological characteristics [5]. Deeper investigation of the underlying mechanisms could help to optimize therapeutic strategies by achieving a balance between glycemic control and arrhythmic risk.

Previous observational studies have yielded conflicting results regarding the impact of glucose‐lowering therapies on arrhythmia risk. Accumulating research suggests that newer antidiabetic drugs, such as glucagon‐like peptide‐1 receptor agonists (GLP‐1RAs), dipeptidyl peptidase‐4 inhibitors (DPP‐4is), and sodium‐glucose cotransporter‐2 inhibitors (SGLT2is), may exert anti‐arrhythmic effects [6, 7, 8, 9]. Nevertheless, several studies have indicated that the anti‐arrhythmic effects of these drugs were not significant, and some even suggested they elevated the risk of arrhythmias [10, 11]. Additionally, previous observational studies focus on the arrhythmogenic effect of sulfonylureas due to potential risk of hypoglycemia [5], whereas other studies failed to find a causal link between sulfonylureas and arrhythmias [12]. Confounding factors, reverse causality and relatively small sample sizes may lead to different results in clinical practice. In addition, complicated biological mechanisms of glucose‐lowering drugs may also obscure the causal relationships between individual drug targets and arrhythmias. Furthermore, increasing evidence linking diabetes to cardiac conduction disorders (i.e., atrioventricular block, left bundle branch block or right bundle branch block), yet whether glucose‐lowering therapies have an impact on these conditions remains largely unexplored, revealing a significant gap in clinical management [13, 14].

Drug‐target Mendelian randomization (MR) is an extension of MR, designed to explore the causal relationship between single drug targets and diseases. By leveraging single nucleotide polymorphisms (SNPs) as instrumental variables (IVs) to proxy drug targets, this method minimizes the confounding factors and reverse causality that may often present in traditional epidemiological studies. Additionally, based on large‐scale genome‐wide association studies (GWASs), drug‐target MR can reduce bias originating from small sample sizes [15]. In our study, we performed a drug‐targeted MR analysis to comprehensively investigate the causal relationship between antidiabetic drug targets and five types of cardiac arrhythmias, including atrial fibrillation (AF), paroxysmal tachycardia (PT), atrioventricular block (AVB), left bundle branch block (LBBB) and right bundle branch block (RBBB).

## 2 Methods

### 2.1 Study Design

**FIGURE 1:**
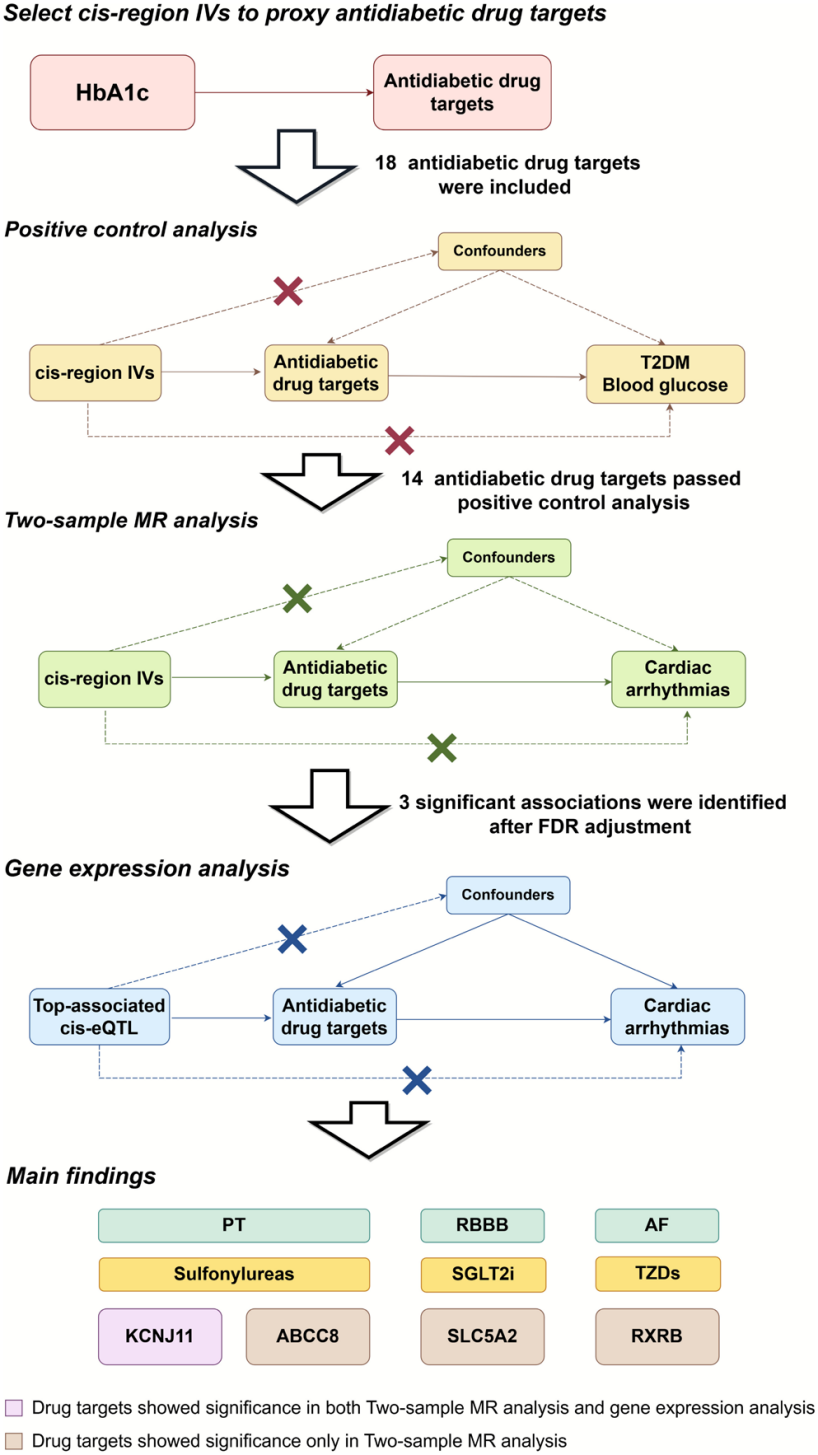
Graphical abstract of our current study. AF, Atrial fibrillation; eQTL, Expression quantitative trait loci; FDR, False discovery rate; HbA1c, Hemoglobin A1C; IVs, Instrumental variables; MR, Mendelian randomization; PT, Paroxysmal tachycardia; RBBB, Right bundle branch block; SGLT2i, Sodium‐glucose cotransporter‐2 inhibitor; T2DM, Type 2 diabetes mellitus; TZDs, Thiazolidinediones. The diagram was created using Figdraw (https://www.figdraw.com).

This drug‐target MR was conducted following the STROBE‐MR (Strengthening the Reporting of Observational Studies in Epidemiologic Trials) statement, and the checklist can be found in Table S1 [16]. The overall design of our current study is illustrated in Figure 1. IVs utilized in our study must satisfy three assumptions: (1) IVs should be robustly associated with exposure; (2) IVs should not be associated with any confounders; (3) IVs should affect the outcomes only through the exposure. All the GWAS used in our study are publicly available, with detailed information provided in Table S2 [15].

### 2.2 Identification of Antidiabetic Drug Targets, Instrument Variables Selection and Positive Controls Analysis

Firstly, the antidiabetic drug targets were derived from the DrugBank pharmacogenetics database (https://go.drugbank.com/). Subsequently, to proxy the exposure of antidiabetic drugs, we selected IVs from the previous GWAS of hemoglobin A1C (HbA1c) across the UK Biobank population as it reflected the long‐term glycemic effect of glucose‐lowering drug targets and has been widely applied in previous drug‐target MR studies (Table S2) [17, 18]. The IVs were extracted within the cis‐window of the targeting gene (±500 kb) and satisfied the criteria of *p* < 5 × 10^−8^, linkage disequilibrium (LD) *r* ^2^ < 0.2, and a clumping window of 10 000 kb [19]. In cases where two neighboring genes shared common IVs due to the overlapping cis‐region, we combined these genes and marked them using a slash, e.g., “ABCC8/KCNJ11.” The F statistic of each SNP was calculated using the formula: *F* = (beta/se)^2^, with an F statistic lower than 10 considered as weak IVs [20]. For positive control analyses, we selected type 2 diabetes mellitus (T2DM) and random blood glucose as positive controls to verify the effectiveness of each antidiabetic drug target (Table S2) [18, 21]. Any drug target significantly associated with T2DM or blood glucose was considered to have passed the positive control.

### 2.3 Data Source of Cardiac Arrhythmias

The GWAS summary statistics of cardiac arrhythmia were derived from the FinnGen R12 datasets (https://r12.finngen.fi/; public release: 4 November 2024) (Table S2). The FinnGen study is a large‐scale genomics initiative that has analyzed over 500 000 Finnish biobank samples and correlated genetic variation with health data to understand disease mechanisms and predispositions [22]. In these comprehensive datasets, we obtained five cardiac arrhythmia phenotypes as outcomes, including AF (63 532 cases, 252 810 controls), PT (12 878 cases, 252 810 controls), AVB (7850 cases, 375 343 controls), LBBB (2766 cases, 375 343 controls), and RBBB (1347 cases, 375 343 controls). Each phenotype was well‐defined according to the ICD‐10 code.

### 2.4 Two‐Sample MR Analysis and Effect Scaling

We applied two‐sample MR analysis to evaluate the causal effects of each antidiabetic drug target on specific types of cardiac arrhythmia. The fixed‐effect inverse variance weighted (IVW) method was applied as the primary approach [23]. This method employs a weighted linear regression model to combine Wald estimates of each SNP to calculate the overall effect size, with the intercept constrained to zero. Additionally, we also performed MR‐Egger and weighted median as supplementary approaches to enhance the reliability of our study [24, 25]. The MR‐Egger method provides a valid effect estimate if there are invalid SNPs as IVs, in violation of the second MR assumption (not via the exposure) [24]. The weighted median method is more robust to outliers than IVW and MR‐Egger, as it utilizes the median ratio estimates of IVW. This method provides consistent estimates if at least 50% of the information comes from potentially valid IVs [25]. The Wald ratio method was applied when only one IV was available for calculation [26]. The effect size in our primary MR analysis was scaled to a per‐standard deviation (SD) reduction in genetically predicted HbA1c levels. Moreover, we also employed random blood glucose to evaluate the estimator of our primary MR analysis. Specifically, we calculated the coefficients (*β*) of the per‐SD decrease in HbA1c on the per‐SD decrease in random blood glucose. We then converted the per‐SD HbA1c decrease into the corresponding per‐SD random blood glucose decrease by multiplying by 1/β, thereby obtaining the OR of a per‐1 SD decrement in random blood glucose on each cardiac arrhythmia [17]. For multiple testing correction, we applied the false discovery rate (FDR) adjustment, with *P*_FDR_ < 0.05 considered as statistically significant.

### 2.5 Sensitivity Analysis

To ensure the robustness of our findings, several sensitivity analyses were conducted. MR‐Egger regression was applied to assess overall horizontal pleiotropy, with a *p* value below 0.05 in the MR‐Egger intercept test indicating the presence of horizontal pleiotropy [24]. Moreover, we also applied the MR‐Pleiotropy RESidual Sum and Outlier (MR‐PRESSO) to detect and correct possible horizontal pleiotropic outliers for significant associations. If the MR‐PRESSO global test yielded a *p* value < 0.05, it indicated the presence of outliers, and the outlier‐corrected effects were subsequently calculated [27]. Cochran's Q statistic was used to evaluate heterogeneity, with a *P* value lower than 0.05 indicating significant heterogeneity [28]. If there was high heterogeneity, the random‐effect IVW method was employed as the primary approach, replacing the fixed‐effect IVW method to counteract potential biases resulting from the heterogeneity. Additionally, we also performed the MR Steiger directional test to assess whether there was reverse causality between antidiabetic drug targets and corresponding diseases. A *p* value of the MR Steiger directional test below 0.05 indicated no evidence of a reverse causal relationship [29].

### 2.6 Gene Expression Analysis

For the marked antidiabetic drug targets identified in our primary MR analysis, we further implemented summary‐data‐based Mendelian randomization (SMR) to explore the effect of their gene expression in blood on the corresponding disease, using expression quantitative trait loci (eQTL) data extracted from the eQTLgen consortium (*n* = 31 684) (Table S2) [30]. Utilizing the top‐associated cis‐QTL, the SMR approach can achieve higher statistical power than the conventional MR method when estimating the causal relationship between QTLs and diseases [31]. Consistent with a previously published article, the top‐associated cis‐QTL in our study was selected within ±1000 kb around the corresponding targeted gene and passed a *p* threshold of 5 × 10^−8^ [32]. The heterogeneity in the dependent instrument (HEIDI) test, which is part of the SMR method, was further conducted to detect whether the observed associations were influenced by LD. A HEIDI test *p* value > 0.05 was considered indicative of no LD.

The two‐sample MR analysis was implemented using the “TwoSampleMR” R package with R version 4.3.2. The SMR and HEIDI analyses were conducted using SMR software (v1.3.1) (https://yanglab.westlake.edu.cn/software/smr/#Overview).

## 3 Results

### 3.1 Identification of Antidiabetic Drug Targets and Positive Control Analysis

**FIGURE 2:**
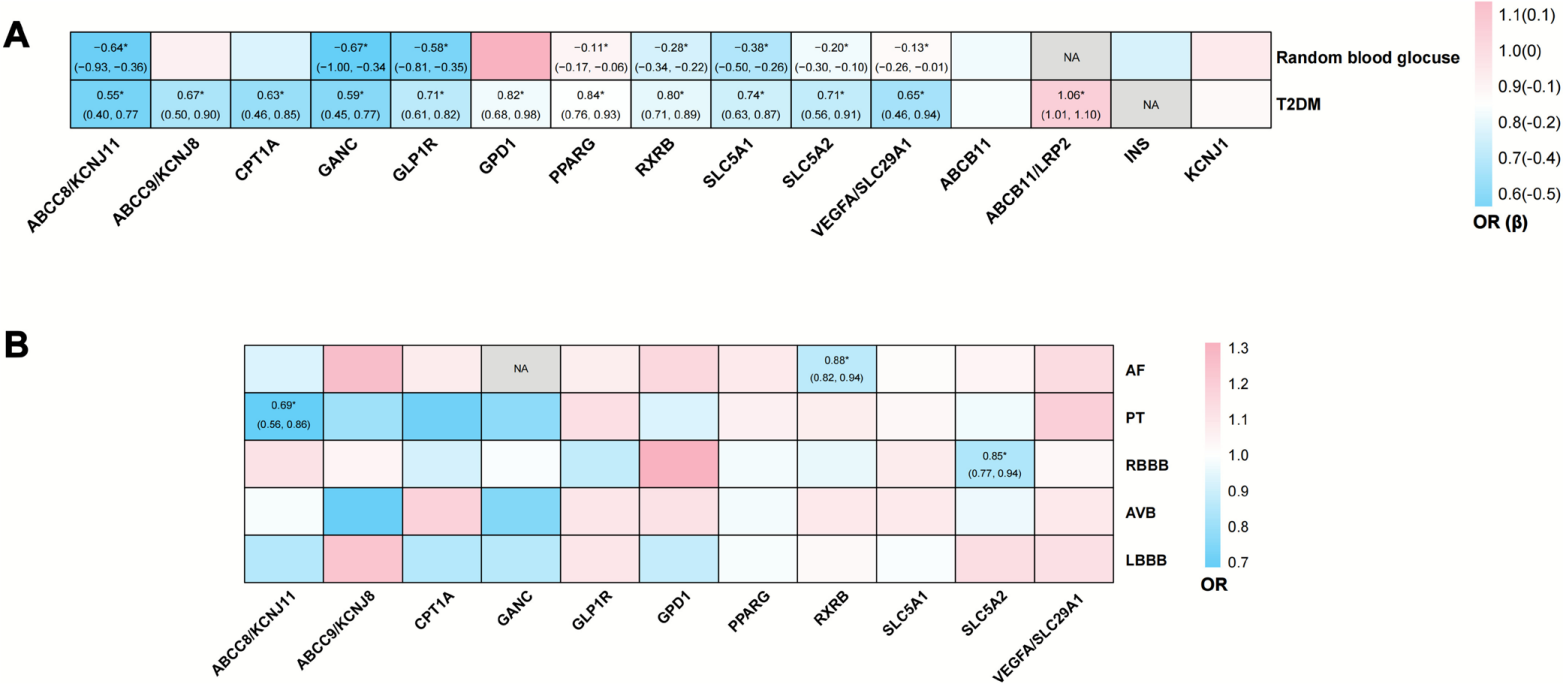
Heatmap of positive control analysis and Two‐sample MR analysis using IVW or Wald ratio. (A) Effect of antidiabetic drug targets on T2DM (estimated by OR) and random blood glucose (estimated by β) in positive control analysis. (B) Effect of antidiabetic drug targets on cardiac arrhythmias (estimated by OR) in Two‐sample MR analysis. NA, Couldn't be tested due to lack of SNP. *Donated the associations with statistical significance after FDR adjustment (*P*_FDR_ < 0.05). AF, Atrial fibrillation; AVB, Atrioventricular block; FDR, False discovery rate; IVW, Inverse variance weighted; LBBB, Left bundle branch block; OR, Odds ratio; RBBB, Right bundle branch block; T2DM, Type 2 diabetes mellitus.

Table 1 presented the detailed information of all the antidiabetic drug targets included in our current study. A total of 140 IVs across 18 antidiabetic drug targets were identified (Table 1; Table S3). The F statistic of each IV comfortably exceeded 10, suggesting no weak instrument bias (Table S3). In the positive control analysis, 14 of 18 antidiabetic drug targets were markedly associated with T2DM and/or random blood glucose, thereby passing the positive control analysis, while the targets ABCB11/LRP2, INS, KCNJ1, and ABCC11 were excluded, as they were not significantly associated with either a reduced risk of T2DM or a lowered random blood glucose (Figure 2A; Table S4).

**TABLE 1:** Summary information of antidiabetic drugs and corresponding target gene included in our study.

Drugs	Target gene	Action	Chromosomal	Position	
				GRCh37_start	GRCh37_end
AGI	GANC	Antagonist	15	42565399	42 645 864
GLP‐1RA	GLP1R	Agonist	6	39016557	39 059 079
Insulin	LRP2	Substrate	2	169983619	170 219 044
Metformin	GPD1	Inhibitor	12	50497791	50 505 096
SGLT2i	SLC5A1	Inhibitor	22	32439248	32 509 016
SGLT2i	SLC5A2	Inhibitor	16	31494444	31 502 090
Sulfonylureas	ABCB11	Inhibitor	2	169777291	169 887 834
Sulfonylureas	ABCC8	Blocker	11	17414045	17 498 392
Sulfonylureas	ABCC9	Modulator	12	21950 323	22 094 360
Sulfonylureas	KCNJ8	Inhibitor	12	21 917 889	21 927 640
Sulfonylureas	KCNJ11	Blocker	11	17 406 795	17 410 893
Sulfonylureas	CPT1A	Inhibitor	11	68 522 088	68 609 384
Sulfonylureas	INS	Regulator	11	2 181 009	2 182 439
Sulfonylureas	KCNJ1	Blocker	11	17 406 795	17 410 893
Sulfonylureas	VEGFA	Other/unknown	6	43 737 946	43 754 224
TZDs	PPARG	Agonist; Regulator	3	12 328 867	12 475 843
TZDs	RXRB	Unknown	6	33 161 365	33 168 630
TZDs	SLC29A1	Inhibitor	6	44 187 352	44 201 879

Abbreviations: AGI, alpha‐glucosidase inhibitors; GLP‐1RA, Glucagon‐like peptide‐1 receptor agonist; SGLT2i, sodium‐glucose cotransporter‐2 inhibitor; TZDs, thiazolidinediones.

### 3.2 Effects of Genetically Predicted Antidiabetic Drug Targets on Cardiac Arrhythmia

**FIGURE 3:**
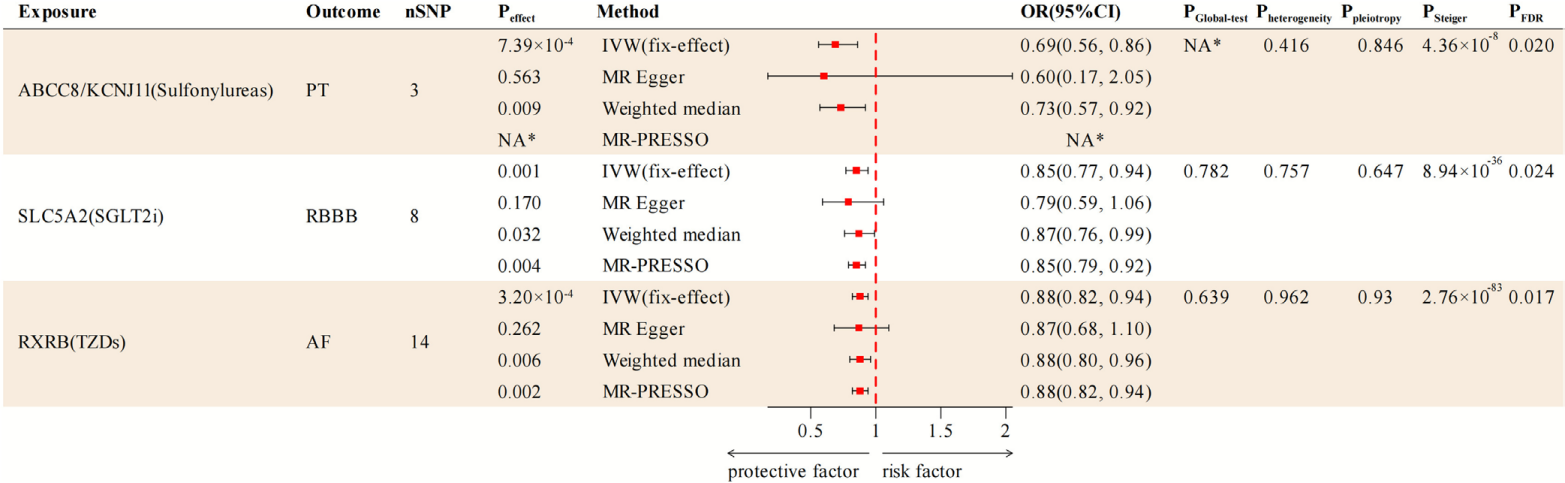
Forest plot of convincing associations between antidiabetic drug targets and arrhythmias in Two‐sample MR analysis (*P*_FDR_ < 0.05). NA*, Couldn't be tested due to lack of enough SNPs. AF, Atrial fibrillation; CI, Confidence interval; FDR, False discovery rate; IVW, Inverse variance weighted; MR‐PRESSO, MR‐Pleiotropy RESidual Sum and Outlier; OR, Odds ratio; PT, Paroxysmal tachycardia; RBBB, Right bundle branch block; SNP, Single nucleotide polymorphism; SGLT2i, Sodium‐glucose cotransporter‐2 inhibitor; TZDs, Thiazolidinediones.

As depicted in Figure 2B, by leveraging the fixed‐effect IVW model as the primary approach, we detected three significant associations after FDR adjustment. Specifically, per 1 SD decrease in HbA1c targeting ABCC8/KCNJ11 of sulfonylureas was significantly linked with a lowered risk of PT (OR: 0.69, 95% CI: 0.56, 0.86, *P*_FDR_ = 0.020) (Figures 2B and 3). Additionally, per 1 SD lowering of HbA1c by SGLT2i targeting SLC5A2 was observed to be associated with a reduced risk of RBBB with statistical significance (OR: 0.85, 95% CI: 0.77, 0.94, *P*_FDR_ = 0.024) (Figures 2B and 3). Moreover, per 1 SD decrement in HbA1c via targeting RXRB of thiazolidinediones (TZDs) was found to be markedly related to a lowered incidence of AF (OR: 0.88, 95% CI: 0.82, 0.94, *P*_FDR_ = 0.017) (Figures 2B and 3). Consistent protective effects were also observed using the supplementary methods (MR‐Egger, weighted median, and MR‐PRESSO), suggesting the robustness of our findings (Figure 3). No significant associations were observed between any antidiabetic drug targets and LBBB or AVB after FDR adjustment (Figure 2B). The detailed information of all MR results and effects scaled by random blood glucose is presented in Table S5.

### 3.3 Sensitivity Analysis

In sensitivity analysis, the MR‐Egger intercept test and Cochran's Q statistic indicated no horizontal pleiotropy and heterogeneity in significant associations, respectively (Figure 3). In addition, the MR Steiger directional test and MR‐PRESSO global test suggested no reverse causality or outliers in significant associations (Figure 3). Collectively, these sensitivity analyses further validate the reliability and robustness of our major findings. Further information on all sensitivity analyses can be found in Table S6.

### 3.4 Gene Expression Analysis

At the gene expression level, we found that the reduced expression of KCNJ11 in blood was significantly associated with a lowered risk of PT (OR: 1.05, 95% CI: 1.01, 1.08, *P*_SMR_ = 0.010). A further HEIDI test showed no LD between KCNJ11 expression and PT (*P*_HEIDI_ = 0.516) (Table 2). This finding is particularly interesting, as the known pharmacological action of sulfonylureas targeting KCNJ11 to lower blood glucose is by inhibiting its expression, which aligns with our key findings (Table S3). No evidence supports a causal link between the expression of SLC5A2 in blood and RBBB (OR: 0.97, 95% CI: 0.89, 1.07, *P*_SMR_ = 0.579) (Table 2). For RXRB expression, no corresponding gene expression data were available in the eQTLgen consortium; therefore, it could not be tested in our study.

**TABLE 2:** Results of gene expression analysis.

Gene	Outcome	Locus	TopSNP	*P*_SMR_	*P*_HEIDI_	OR (95% CI)
KCNJ11	PT	11:17409142	rs2074310	0.010	0.516	1.05 (1.01, 1.08)
SLC5A2	RBBB	16:31498252	rs6565236	0.579	0.142	0.97 (0.89, 1.07)

Abbreviations: CI, confidence interval; HEIDI, heterogeneity in the dependent instrument; OR, odds ratio; PT, paroxysmal tachycardia; RBBB, right bundle branch block; SMR, summary‐data‐based Mendelian randomization; SNP, single nucleotide polymorphism.

## 4 Discussion

In our current research, we systematically evaluated the causal effect of antidiabetic drug targets on cardiac arrhythmias and identified three robust associations after FDR adjustment. Specifically, the glucose‐lowering targets KCNJ11/ABCC8 (sulfonylureas), SLC5A2 (SGLT2i), and RXRB (TZDs) were associated with a decreased risk of PT, AF and RBBB, respectively. Moreover, gene expression analysis further supported the protective role of KCNJ11 in PT. Our findings extend beyond the scope of previous studies, providing novel insights into the causal relationship between antidiabetic drug targets and cardiac arrhythmias.

Recently, the association between glucose homeostasis and cardiac arrhythmias has drawn widespread attention [13, 33]. In the setting of T2DM, chronic hyperglycemia, systemic inflammation, and oxidative stress may individually and additively contribute to myocardial fibrosis in the atria, ventricles, and conduction system, thereby increasing the incidence of cardiac arrhythmias. Besides, T2DM is associated with autonomic neuropathy and electrolyte abnormalities, which result in increased risk of electrical disorders [3, 34, 35]. These findings highlight the importance of optimal glycemic control in preventing arrhythmias and reducing the burden of cardiovascular complications in T2DM.

Sulfonylureas are currently the most commonly used medications (add‐on to metformin) for the treatment of diabetes due to their reliable glucose‐lowering effects, low cost, and demonstrated ability to reduce microvascular complications [36]. These drugs primarily exert their glucose‐lowering effects by blocking the ATP‐sensitive potassium (K_ATP_) channels on the membrane of pancreatic β cells, stimulating insulin secretion, thereby lowering blood glucose levels and controlling the progression of diabetes [37]. The K_ATP_ channels are assembled from the Kir6.2 and SUR1 subunits in a 4:4 stoichiometry, encoded by the KCNJ11 and ABCC8 genes located adjacently on human chromosome 11p15.1. Notably, these K_ATP_ channels are also expressed on the surface of myocardium, which may particularly mediate the effects of sulfonylureas on the heart, particularly influencing the electrophysiological properties of cardiomyocytes [38]. Mechanistically, previous preclinical studies have shown that blocking cardiac cell membrane K_ATP_ channels during myocardial ischemia reduces potassium efflux and increases action potential duration, thereby attenuating lethal arrhythmias [37]. However, these basic studies exhibited significant heterogeneity due to variations in study designs and methods, limiting the further generalization of these conclusions [39]. Additionally, there is still a lack of studies conducted in humans. Though previous clinical studies suggest that sulfonylureas might reduce the incidence of arrhythmias by blocking KCNJ11, these studies have been constrained to the context of structural heart disease and ischemic conditions, with limited long‐term follow‐up [40]. In our study, we are the first to reveal that targeting KCNJ11/ABCC8 for diabetes treatment could reduce the incidence of PT from a genetic perspective, with gene expression analysis further supporting the role of KCNJ11 in PT. This finding highlights the anti‐arrhythmic effects of sulfonylureas and fills a gap in current research. Importantly, due to the nature of MR, this effect is lifelong and unaffected by various disease states, effectively addressing the issues present in previous studies.

Meanwhile, we also revealed that targeting SLC5A2 of SGLT2i to lower blood glucose was significantly associated with a reduced occurrence of RBBB. RBBB is characterized by either a significant delay or a lack of electrical conduction through the right bundle branch and distal Purkinje fibers and is common in populations with a prevalence ranging from 0.2% to 1.3% [41]. From a pathophysiological perspective, the development of RBBB is typically associated with fibrosis of the cardiac conduction system, ischemic injury, ventricular remodeling, and abnormalities in ion channel function [42]. Of note, preliminary clinical studies conducted by Gaba et al. indicated that the occurrence of RBBB was associated with an elevated risk of all‐cause mortality, cardiovascular‐related mortality, and reduced exercise tolerance [43]. Hence, given the detrimental impact of RBBB on cardiovascular outcomes, exploring potential therapeutic strategies to mitigate its progression is of great clinical significance. SGLT2is are promising antidiabetic drugs, demonstrating significant blood glucose control while also improving cardiovascular and renal outcomes [44]. Mechanistically, several fundamental studies have suggested that SGLT2i may enhance cardiac Na^+^‐Ca^2+^ homeostasis, autophagy balance, and mitochondrial function [45]. These pathways collectively reduce cardiac structural remodeling and inflammation, potentially playing a role in preventing the progression and development of RBBB. However, to date, there is still a lack of studies unveiling the underlying mechanisms linking SGLT2is to RBBB. Hence, though our findings suggest that SGLT2is may emerge as a potential therapeutic option to improve the prognosis of patients with RBBB, further basic research is required to unveil the pathophysiological mechanisms and long‐term effects of SGLT2is on conduction system cells and their role in preventing or reversing RBBB.

Retinoid X Receptor β (RXRB) is a member of the retinoid X receptor family, which belongs to the nuclear receptor superfamily and functions as a transcription factor [46]. As an obligate heterodimeric partner for peroxisome proliferator‐activated receptor gamma (PPARG), the primary target of TZDs, RXRB has been considered a potential target for blood glucose regulation [46, 47]. Previous studies have shown that TZDs reduce the risk of AF, but the role of RXRB in this effect remains unclear. In our current study, we uncovered a causal relationship between RXRB and a reduced risk of AF. However, the association between the target PPARG and AF was not statistically significant (*p* = 0.08). Based on these findings, we hypothesize that the benefits of TZDs in reducing AF incidence may be primarily mediated by RXRB, which is located downstream of PPARG, rather than by PPARG itself. Interestingly, in addition to its involvement in glucose metabolism, recent studies have also highlighted RXRB's role in lipid metabolism and the inflammatory response, both of which are closely related to AF [48, 49]. Therefore, further research is needed to fully elucidate the underlying mechanisms linking RXRB to AF risk.

### 4.1 Limitations

Our study has several limitations. First, all the GWAS data used in our current research are derived from European populations. Therefore, when generalizing our findings to populations of other ancestries, potential population biases between different ancestries should be considered. Second, due to the limitations of the original GWAS data, we were unable to further explore the association between KCNJ11/ABCC8 and subtypes of PT (e.g., ventricular tachycardia, supraventricular tachycardia). Third, as the SMR analysis relied on eQTL data derived from whole blood, the findings may not fully capture cardiac tissue–specific transcriptional regulation. Future studies employing eQTL data from heart tissues are required to confirm and extend our results. Fourth, variations in drug formulations, administration methods, inter‐individual differences in metabolism, and the binding affinity of drugs to their intended targets in real‐world settings mean that the effect sizes estimated from our MR analysis may not fully reflect the actual therapeutic benefits of these drugs in clinical practice. Fifth, the precise underlying mechanisms connecting identified antidiabetic drug targets to arrhythmia are not fully established. Collectively, these limitations highlight the need for further experimental and clinical research to unveil the underlying mechanism, address potential population biases, explore additional arrhythmia subtypes, and validate our findings in larger, more diverse cohorts.

## 5 Conclusion

Our research suggests a protective role of KCNJ11/ABCC8 (sulfonylureas), SLC5A2 (SGLT2i), and RXRB (TZDs) in preventing PT, RBBB, and AF, respectively. These findings offer novel insights into the interrelationship between antidiabetic drug targets and cardiac arrhythmias.

## Author Contributions

Z.‐Q.S. and Y.‐H.C. designed this study. Z.‐Q.S., Z.‐B.Z. and S.‐K.W. wrote the original manuscript and analyzed the data. S.‐Y.F., R.‐F.S. and Y.‐H.C. revised the manuscript. Z.‐B.Z., B.‐X.W. and Y.‐H.S. verified the correctness of the data.

## Funding

This work was supported by the Natural Science Foundation of China (NSFC) Grant (No. 81900229) to Yi‐He Chen.

## Ethics Statement

All summarized statistics used in this MR analyses were generated by previous studies, for which ethical approval and individual consent could be found in the corresponding original GWAS article.

## Consent

The authors have nothing to report.

## Conflicts of Interest

The authors declare no conflicts of interest.

## Supporting information


**Data S1:** joa370241‐sup‐0001‐DataS1.pdf.

**Table S1:** Self‐inspection results of STROBE‐MR checklist.
**Table S2:** Detailed information of data source used in our study.
**Table S3:** Detailed information of SNPs and corresponding effects on HbA1c that used for each drug target.
**Table S4:** Detailed information of positive control analysis.
**Table S5:** Detailed information of all results in two‐sample MR analysis and effect scaled by random blood glucose.
**Table S6:** Detailed information of all sensitivity results in two‐sample MR analysis.

## Data Availability

All the data used in our research is publicly available. The download links are listed in Table S2.
