# Supplementary material for: Exploring the Therapeutic Potential of Antidiabetic Drugs in Cardiac Arrhythmia Management: A Drug Target Mendelian Randomization Study
Source: J Arrhythm. 2025 Dec 4;41(6):e70241. doi: 10.1002/joa3.70241 (PMC12678633; doi:10.1002/joa3.70241)
Supplement: Supplementary file 1 — Data S1: joa370241‐sup‐0001‐DataS1.pdf. [file JOA3-41-e70241-s002.pdf]

WILEY

# Request for Changes to Journal Article Author List

## What is this form for?

- Use this form to add or remove authors from an article.
- This form is not to request a post-publication change to an author's name (for example, to correct a misspelling). Please contact the Production Editor directly to make arrangements for these changes.
- This form is for use at all stages of submission and publication. **Change requests will not be considered unless the fully filled form is submitted.**

## What should authors be aware of before completing and submitting this form?

- Requests to change an article's author list will be carefully reviewed by the Publisher and Editor, in line with the authors and contributors policies outlined in Wiley's Best Practice Guidelines on Research Integrity and Publishing Ethics, by Committee on Publication Ethics guidance, and in the journal's Author Guidelines available on Wiley Online Library.
- If your article has already been published, a change to the online Version of Record will require an enduring erratum/correction statement.
- The initiating author must provide to each involved party every page of this document, including this cover page, and any additional pages to be attached, for review.
- No co-author (those remaining, those newly added, those to be removed) or individuals and/or representatives of multi-author collaborative or consortia groups **should sign this form without reading** a) every page of this form in full including the cover page and any attachments, b) the relevant authorship and contributorship policies outlined on the cover page, and c) agreeing with all changes to the author list proposed herein.
- The Editor and Publisher are not empowered to arbitrate authorship disputes; in the event of an authorship dispute, consult your institution's research ethics department for guidance.

## What will be required to complete this form?

- The undertaking—confirmed by way of a signature—of each and every involved party (those remaining, those newly added, those to be removed) that the requested change accurately reflects the authorship of the article at hand.
- A brief explanation of why the requested change is needed.

## How do I submit this form and what will happen next?

- Submit the completed form to the journal's editorial or production office (visit the journal's homepage on Wiley Online Library to find the email address); incomplete forms will not be considered.
- Note that the below tables are in editable PDF format; we recommend completing the details using this functionality. If you are unable to use this functionality and require additional space, provide numbered attached documents with your request that clearly show the changes being requested.

- All authors (including any added or removed) should be copied on the email requesting the change.
- Wiley accepts handwritten signatures and e-signatures (e-signatures should preferably be provided using DocuSign, https://go.docusign.com; more information can be found at https://support.docusign.com/en/knowledgeSearch?by=topic&topic=sign_documents&category=sign). If authors are unable to sign on a single form, multiple versions of the same form, collated into a single PDF, will be acceptable.
- The Publisher and Editor may seek further information from each author on the requested change, if appropriate.
- The Publisher and Editor may seek further information from each author's institution on the requested change, if appropriate.

**Table 1. Article Information and change request**

| | |
|---|---|
| **Article title** | ntial of antidiabetic drugs in cardiac arrhythmia management: A drug target Mendelian Randomization study |
| **Journal title** | Journal of Arrhythmia |
| **Submission ID or DOI** | JOA-2025-0478 |
| **Provide a brief explanation of and reason for the changes requested** | Suyin Feng and Runfeng Sun provided the guidance for revising the manuscript |

**Table 2. Provide the complete and correct author list, in order, as it should appear on the article (attach an additional sheet if more space is needed)**

| Author Name** | | Degree/s (e.g. BSc, PhD) | Corr. author (Y/N) | Institutional affiliation | Email address | ORCID iD or Scopus Author ID | Signature* | Date |
|---|---|---|---|---|---|---|---|---|
| Given Name† | Family Name | | | | | | | |
| Zheng Qi | Song | MD | N | 'enzhou, Zhejiang, China. | hengqi@wmu.edu.cn | NA | Zheng Qi Song | 28-Oct-20 |
| Zhi Bo | Zhou | MD | N | 'enzhou, Zhejiang, China. | 368770772@163.com | NA | Zhi Bo Zhou | 28-Oct-20 |
| Bo Xiang | Wang | MD | N | 'enzhou, Zhejiang, China. | 588995075@163.com | NA | BoXiang Wang | 28-Oct-20 |
| Sheng Ke | Wu | MD | N | 'enzhou, Zhejiang, China. | wsk171016@163.com | NA | Sheng Ke Wu | 28-Oct-20 |
| Yi Han | Sun | MD | N | niversity,Wenzhou,China. | .140228@wmu.edu.cn | NA | YiHang Sun | 28-Oct-20 |
| Yi He | Chen | MD | Y | 'enzhou, Zhejiang, China. | henyihe@wmu.edu.cn | NA | YiHe Chen | 28-Oct-20 |
| Suyin | Feng | MD | Y | ng Medical University); | suyin@njmu.edu.cn | NA | Suyin Feng | 28-Oct-20 |
| Runfeng | Sun | MD | Y | ng Medical University); | ng_dh@njmu.edu.cn | NA | Runfeng Sun | 28-Oct-20 |
| | | | | | | | | |

*By signing Table 2, individuals and/or representatives of multi-author collaborative or consortia groups confirm that the author list shown here accurately reflect the authorship of the article cited in Table 1

†Include the author's middle initials with the given name, if applicable

**In cases of multi-author collaborative or consortia groups the most appropriate representative or legal guarantor must identify themselves and sign on behalf of the group



No co-author (those remaining, those newly added, those to be removed) or individuals and/or representatives of multi-author collaborative or consortia groups **should sign this form without reading** a) every page of this form in full including the cover page and any attachments, b) the relevant authorship and contributorship policies outlined on the cover page, and c) agreeing with all changes to the author list proposed herein.

**Table 3. Provide the following information for each NEWLY ADDED author in Table 2 (leave blank if no new authors have been added)**

| Author Name** | | Conflict of Interest statement | Acknowledgements | Contribution statement†† |
|---|---|---|---|---|
| Given Name | Family Name | | | |
| Suyin | Feng | None | We thank Su-Yin Feng, Run-Feng Sun and Yi-He Chen for assisting with the guiding and revising of the manuscript. | S-Y Feng, R-F Sun and Y-H Chen revised the manuscript. |
| Runfeng | Sun | None | We thank Su-Yin Feng, Run-Feng Sun and Yi-He Chen for assisting with the guiding and revising of the manuscript. | S-Y Feng, R-F Sun and Y-H Chen revised the manuscript. |
| | | | | |
| | | | | |
| | | | | |

**In cases of multi-author collaborative or consortia groups the most appropriate representative or legal guarantor must identify themselves and sign on behalf of the group
††Ensure the contribution statement aligns with the journal's contribution role taxonomy (e.g. CRediT), if relevant. This information can be found on the journal's Author Guidelines page on Wiley Online Library.



No co-author (those remaining, those newly added, those to be removed) or individuals and/or representatives of multi-author collaborative or consortia groups **should sign this form without reading** a) every page of this form in full including the cover page and any attachments, b) the relevant authorship and contributorship policies outlined on the cover page, and c) agreeing with all changes to the author list proposed herein.

WILEY

**Table 4. Provide a list of the individuals and/or representatives of multi-author collaborative or consortia groups to be REMOVED from the author list as it was originally provided for the article cited in Table 1 (leave blank if no authors are to be removed)**

| Author Name** | | Institutional affiliation | Email address | Signature* | Date |
|---|---|---|---|---|---|
| Given Name† | Family Name | | | | |
| | | | | | |
| | | | | | |
| | | | | | |

*By signing Table 4, individuals and/or representatives of multi-author collaborative or consortia groups confirm that the author list shown in Table 2 accurately reflect the authorship of the article cited in Table 1
†Include the author's middle initials with the first name, if applicable
**In cases of multi-author collaborative or consortia groups the most appropriate representative or legal guarantor must identify themselves and sign on behalf of the group



No co-author (those remaining, those newly added, those to be removed) or individuals and/or representatives of multi-author collaborative or consortia groups **should sign this form without reading** a) every page of this form in full including the cover page and any attachments, b) the relevant authorship and contributorship policies outlined on the cover page, and c) agreeing with all changes to the author list proposed herein.
