# Supplementary material for: Exploring the Therapeutic Potential of Antidiabetic Drugs in Cardiac Arrhythmia Management: A Drug Target Mendelian Randomization Study
Source: J Arrhythm. 2025 Dec 4;41(6):e70241. doi: 10.1002/joa3.70241 (PMC12678633; doi:10.1002/joa3.70241)
Supplement: Supplementary file 2 — Table S1: Self‐inspection results of STROBE‐MR checklist. Table S2: Detailed information of data source used in our study. Table S3: Detailed information of SNPs and corresponding effects on HbA1c that used for each drug target. Table S4: Detailed information of positive control analysis. Table S5: Detailed information of all results in two‐sample MR analysis and effect scaled by random blood glucose. Table S6: Detailed information of all sensitivity results in two‐sample MR analysis. [file JOA3-41-e70241-s001.docx]

| **Legend** |
| --- |
| Table S1.Self-inspection results of STROBE-MR checklist |
| Table S2. Detailed information of data source used in our study |
| Table S3. Detailed information of SNPs and corresponding effects on HbA1c that used for each drug target. |
| Table S4. Detailed information of positive control analysis |
| Table S5. Detailed information of all results in two-sample MR analysis and effect scaled by random blood glucose |
| Table S6. Detailed information of all sensitivity results in two-sample MR analysis |

| **Table S1.Self-inspection results of STROBE-MR checklist** | | | |
| --- | --- | --- | --- |
| **Item No.** | **Section** | **Checklist item** | **Relevant text from manuscript** |
| 1 | **TITLE and ABSTRACT** | Indicate Mendelian randomization (MR) as the study’s design in the title and/or the abstract if that is a main purpose of the study | Exploring the therapeutic potential of antidiabetic drugs in cardiac arrhythmia management: A drug target Mendelian Randomization study |
|  | **INTRODUCTION** |  |  |
| 2 | **Background** | Explain the scientific background and rationale for the reported study. What is the exposure? Is a potential causal relationship between exposure and outcome plausible? Justify why MR is a helpful method to address the study question | Introduction: paragraphs 1-3 |
|  |  |  |  |
|  |  |  |  |
|  |  |  |  |
| 3 | **Objectives** | State specific objectives clearly, including pre-specified causal hypotheses (if any). State that MR is a method that, under specific assumptions, intends to estimate causal effects | Introduction: paragraphs 3 |
|  |  |  |  |
| **METHODS** |  |  |  |
| **4** | **Study design and data sources** | Present key elements of the study design early in the article. Consider including a table listing sources of data for all phases of the study. For each data source contributing to the analysis, describe the following: |  |
|  | a) | Setting: Describe the study design and the underlying population, if possible. Describe the setting, locations, and relevant dates, including periods of recruitment, exposure, follow-up, and data collection, when available. | Method: Study Design |
|  | b) | Participants: Give the eligibility criteria, and the sources and methods of selection of participants. Report the sample size, and whether any power or sample size calculations were carried out prior to the main analysis | Method：Identification of antidiabetic drug targets, instrument variables selection and positive controls analysis; Data source of cardiac arrhythmias and Supplementary Table 2 |
|  | c) | Describe measurement, quality control and selection of genetic variants | Method: Identification of antidiabetic drug targets, instrument variables selection and positive controls analysis |
|  | d) | For each exposure, outcome, and other relevant variables, describe methods of assessment and diagnostic criteria for diseases | Method：Identification of antidiabetic drug targets, instrument variables selection and positive controls analysis; Data source of cardiac arrhythmias and Supplementary Table 2 |
|  | e) | Provide details of ethics committee approval and participant informed consent, if relevant | Method：Data source of cardiac arrhythmias and Supplementary Table 2 |
| 5 | **Assumptions** | Explicitly state the three core IV assumptions for the main analysis (relevance, independence and exclusion restriction) as well assumptions for any additional or sensitivity analysis | Method: Study Design |
|  |  |  |  |
| 6 | **Statistical methods: main analysis** | Describe statistical methods and statistics used |  |
|  | a) | Describe how quantitative variables were handled in the analyses (i.e., scale, units, model) | Method: Two-sample MR analysis and effect scaling |
|  |  |  |  |
|  | b) | Describe how genetic variants were handled in the analyses and, if applicable, how their weights were selected | Method：Two-sample MR analysis and effect scaling |
|  | c) | Describe the MR estimator (e.g. two-stage least squares, Wald ratio) and related statistics. Detail the included covariates and, in case of two-sample MR, whether the same covariate set was used for adjustment in the two samples | Method：Two-sample MR analysis and effect scaling |
|  | d) | Explain how missing data were addressed | Not applicable |
|  | e) | If applicable, indicate how multiple testing was addressed | Method：Two-sample MR analysis and effect scaling |
| 7 | **Assessment of assumptions** | Describe any methods or prior knowledge used to assess the assumptions or justify their validity | Method: Sensitivity analysis and Gene expression analysis |
|  |  |  |  |
| 8 | **Sensitivity analyses and additional analyses** | Describe any sensitivity analyses or additional analyses performed (e.g. comparison of effect estimates from different approaches, independent replication, bias analytic techniques, validation of instruments, simulations) | Method: Sensitivity analysis and Gene expression analysis |
| 9 | **Software and pre-registration** |  |  |
|  | a) | Name statistical software and package(s), including version and settings used | The two-sample MR analysis was implemented using the "TwoSampleMR" R package with R version 4.3.2. The SMR and HEIDI analyses were conducted using SMR software (v1.3.1) (https://yanglab.westlake.edu.cn/software/smr/#Overview). |
|  | b) | State whether the study protocol and details were pre-registered (as well as when and where) | No Applicable |
|  | **RESULTS** |  |  |
| 10 | **Descriptive data** |  |  |
|  | a) | Report the numbers of individuals at each stage of included studies and reasons for exclusion. Consider use of a flow diagram | Method: Data source of cardiac arrhythmias; Figure 1 |
|  | b) | Report summary statistics for phenotypic exposure(s), outcome(s), and other relevant variables (e.g. means, SDs, proportions) | Data source of cardiac arrhythmias; Table S2 |
|  |  |  |  |
|  | c) | If the data sources include meta-analyses of previous studies, provide the assessments of heterogeneity across these studies | No Applicable |
|  | d) | For two-sample MR: | No Applicable |
|  |  | i. Provide justification of the similarity of the genetic variant-exposure associations between the exposure and outcome samples |  |
|  |  | ii. Provide information on the number of individuals who overlap between the exposure and outcome studies |  |
| 11 | **Main results** |  |  |
|  | a) | Report the associations between genetic variant and exposure, and between genetic variant and outcome, preferably on an interpretable scale | Restlts: Identification of antidiabetic drug targets and positive control analysis; Supplementary Table S3 |
|  |  |  |  |
|  | b) | Report MR estimates of the relationship between exposure and outcome, and the measures of uncertainty from the MR analysis, on an interpretable scale, such as odds ratio or relative risk per SD difference | Results:Effects of genetically predicted antidiabetic drug targets on cardiac arrhythmia; Suplementary Table S4 and Supplementary Table S5 |
|  |  |  |  |
|  | **c)** | **If relevant, consider translating estimates of relative risk into absolute risk for a meaningful time period** |  |
|  | d) | Consider plots to visualize results (e.g. forest plot, scatterplot of associations between genetic variants and outcome versus between genetic variants and exposure) | Figure 2 and Figure 3 |
| 12 | **Assessment of assumptions** |  |  |
|  | a) | Report the assessment of the validity of the assumptions by removing confounders-related SNPs. | Restlts: Sensitivity analysis and Spupplementary Table S6 |
|  | b) | Report any additional statistics (e.g., assessments of heterogeneity across genetic variants, such as I2, Q statistic or E-value) | Results :Gene expression analysis and Table 2 |
| 13 | **Sensitivity analyses and additional analyses** |  |  |
|  | a) | Report any sensitivity analyses to assess the robustness of the main results to violations of the assumptions | Results: Sensitivity analysis and Supplementary Table S6 |
|  |  |  |  |
|  | b) | Report results from other sensitivity analyses or additional analyses | Results :Gene expression analysis and Table 2 |
|  | c) | Report any assessment of direction of causal relationship (e.g., bidirectional MR) | Results: Sensitivity analysis and Supplementary Table S6 |
|  |  |  |  |
|  | d) | When relevant, report and compare with estimates from non-MR analyses | No Applicable |
|  |  |  |  |
|  | e) | Consider additional plots to visualize results (e.g., leave-one-out analyses) | No Applicable |
|  | **DISCUSSION** |  |  |
| 14 | **Key results** | Summarize key results with reference to study objectives | Discussion: paragraph 1 |
| 15 | **Limitations** | Discuss limitations of the study, taking into account the validity of the IV assumptions, other sources of potential bias, and imprecision. Discuss both direction and magnitude of any potential bias and any efforts to address them | Discussion: Limitations |
| 16 | **Interpretation** |  |  |
|  | a) | Meaning: Give a cautious overall interpretation of results in the context of their limitations and in comparison with other studies | Discussion: paragraphs 2-5 |
|  |  |  |  |
|  |  |  |  |
|  | b) | Mechanism: Discuss underlying biological mechanisms that could drive a potential causal relationship between the investigated exposure and the outcome, and whether the gene-environment equivalence assumption is reasonable. Use causal language carefully, clarifying that IV estimates may provide causal effects only under certain assumptions |  |
|  |  |  |  |
|  | c) | Clinical relevance: Discuss whether the results have clinical or public policy relevance, and to what extent they inform effect sizes of possible interventions |  |
|  |  |  |  |
| 17 | **Generalizability** | Discuss the generalizability of the study results (a) to other populations, (b) across other exposure periods/timings, and (c) across other levels of exposure |  |
|  |  |  |  |
|  |  |  |  |
|  | **OTHER INFORMATION** |  |  |
| 18 | **Funding** | Describe sources of funding and the role of funders in the present study and, if applicable, sources of funding for the databases and original study or studies on which the present study is based | Declarations: Source of funding |
| 19 | **Data and data sharing** | Provide the data used to perform all analyses or report where and how the data can be accessed, and reference these sources in the article. Provide the statistical code needed to reproduce the results in the article, or report whether the code is publicly accessible and if so, where | Declarations: Data availability |
| 20 | **Conflicts of Interest** | All authors should declare all potential conflicts of interest | Declarations: Competing interests |

| **Table S2. Detailed information of data source used in our study** | | | | | | | | | |
| --- | --- | --- | --- | --- | --- | --- | --- | --- | --- |
| **Phenotype** | **Resource** | **Type** | **Sample Size** | **Case/Control** | **Population** | **PubMed ID** | **IEU GWAS ID** | **Download link** | **Statistical analysis** |
| HbA1c | UK Biobank | continuous phenotype | 389,889 | NA | European | 34017140 | ebi-a-GCST90014006 | [https://gwas.mrcieu.ac.uk/datasets/ebi-a-GCST90014006/](https://gwas.mrcieu.ac.uk/datasets/ebi-a-GCST90014006/) | Select IVs for drug targets in two-sample MR analysis |
| eQTLs | eQTLGen | continuous phenotype | 31,684 | NA | Predominantly European | 34475573 | eqtl-a | <https://www.eqtlgen.org/> | Provide gene expression data in gene expression analysis |
| Random blood glucose | UK Biobank | continuous phenotype | 357,580 | NA | European | 34017140 | ebi-a-GCST90014005 | <https://gwas.mrcieu.ac.uk/datasets/ebi-a-GCST90014005/> | Positive control in MR, scale the effects from HbA1c to glucose |
| Type 2 diabetes mellitus | DIAGRAM, GERA, and UKB | binary phenotype | 659,316 | 62,892 cases, 596,424 controls | Predominantly European | 30054458 | ebi-a-GCST006867 | <https://gwas.mrcieu.ac.uk/datasets/ebi-a-GCST006867/> | Positive control in MR |
| Atrial fibrillation | FinnGen R12 | binary phenotype | 316,342 | 63,532 cases, 252,810 controls | European | 36653562 | NA | [https://r12.finngen.fi/](https://r12.finngen.fi/) | Outcomes in two-sample MR analysis and SMR analysis |
| Paroxysmal tachycardia |  | binary phenotype | 265,688 | 12,878 cases, 252,810 controls | European |  |  |  |  |
| Atrioventricular block |  | binary phenotype | 383,193 | 7,850 cases, 375,343 controls | European |  |  |  |  |
| Left bundle branch block |  | binary phenotype | 378,109 | 2,766 cases, 375,343 controls | European |  |  |  |  |
| Right bundle branch block |  | binary phenotype | 376,690 | 1,347 cases, 375,343 controls | European |  |  |  |  |

| **Table S3. Detailed information of SNPs and corresponding effects on HbA1c that used for each drug target.** | | | | | | | | | | | | |
| --- | --- | --- | --- | --- | --- | --- | --- | --- | --- | --- | --- | --- |
| **Target Gene** | **Drug** | **SNP** | **chr** | **pos** | **effect allele** | **other allele** | **beta** | **se** | **pval** | **F statistic** | **phenotype** | **samplesize** |
| ABCB11 | Sulfonylureas | rs114388308 | 2 | 169355957 | T | C | -0.031 | 0.005 | 6.37E-10 | 38.2 | HbA1c | 389889 |
| ABCB11 | Sulfonylureas | rs183034862 | 2 | 169313518 | T | C | 0.056 | 0.007 | 6.51E-17 | 69.8 | HbA1c | 389889 |
| ABCB11 | Sulfonylureas | rs2601091 | 2 | 169295677 | G | A | 0.025 | 0.004 | 4.14E-10 | 39.0 | HbA1c | 389889 |
| ABCB11 | Sulfonylureas | rs79467716 | 2 | 169404334 | A | G | -0.055 | 0.008 | 3.23E-11 | 44.0 | HbA1c | 389889 |
| ABCB11/LRP2 | Sulfonylureas/Insulin | rs10206400 | 2 | 169730872 | A | C | -0.048 | 0.002 | 6.40E-108 | 487.0 | HbA1c | 389889 |
| ABCB11/LRP2 | Sulfonylureas/Insulin | rs112308555 | 2 | 169976474 | A | T | 0.032 | 0.006 | 4.15E-08 | 30.1 | HbA1c | 389889 |
| ABCB11/LRP2 | Sulfonylureas/Insulin | rs114764002 | 2 | 169776141 | T | A | -0.087 | 0.006 | 9.60E-54 | 238.2 | HbA1c | 389889 |
| ABCB11/LRP2 | Sulfonylureas/Insulin | rs115128825 | 2 | 169748833 | A | C | 0.072 | 0.007 | 6.71E-24 | 101.6 | HbA1c | 389889 |
| ABCB11/LRP2 | Sulfonylureas/Insulin | rs115941163 | 2 | 169811749 | T | C | 0.085 | 0.009 | 3.65E-21 | 89.2 | HbA1c | 389889 |
| ABCB11/LRP2 | Sulfonylureas/Insulin | rs116748035 | 2 | 169537003 | A | G | -0.051 | 0.007 | 1.77E-14 | 58.8 | HbA1c | 389889 |
| ABCB11/LRP2 | Sulfonylureas/Insulin | rs13016739 | 2 | 169719362 | A | G | 0.062 | 0.008 | 9.11E-15 | 60.1 | HbA1c | 389889 |
| ABCB11/LRP2 | Sulfonylureas/Insulin | rs13430620 | 2 | 169768891 | C | A | -0.073 | 0.005 | 4.54E-51 | 226.0 | HbA1c | 389889 |
| ABCB11/LRP2 | Sulfonylureas/Insulin | rs145353824 | 2 | 169765277 | C | A | 0.104 | 0.011 | 1.93E-21 | 90.4 | HbA1c | 389889 |
| ABCB11/LRP2 | Sulfonylureas/Insulin | rs147001976 | 2 | 169639844 | A | G | -0.039 | 0.006 | 5.69E-12 | 47.4 | HbA1c | 389889 |
| ABCB11/LRP2 | Sulfonylureas/Insulin | rs148766104 | 2 | 169912240 | A | G | -0.048 | 0.008 | 2.16E-10 | 40.3 | HbA1c | 389889 |
| ABCB11/LRP2 | Sulfonylureas/Insulin | rs150729386 | 2 | 169778608 | A | C | -0.054 | 0.005 | 3.61E-27 | 116.5 | HbA1c | 389889 |
| ABCB11/LRP2 | Sulfonylureas/Insulin | rs16855893 | 2 | 169652647 | C | T | 0.038 | 0.005 | 4.94E-16 | 65.8 | HbA1c | 389889 |
| ABCB11/LRP2 | Sulfonylureas/Insulin | rs16856252 | 2 | 169789720 | C | T | 0.047 | 0.003 | 6.42E-47 | 206.9 | HbA1c | 389889 |
| ABCB11/LRP2 | Sulfonylureas/Insulin | rs17251018 | 2 | 169588195 | G | A | -0.014 | 0.002 | 5.30E-11 | 43.1 | HbA1c | 389889 |
| ABCB11/LRP2 | Sulfonylureas/Insulin | rs17251700 | 2 | 169613639 | A | G | -0.049 | 0.007 | 8.18E-14 | 55.8 | HbA1c | 389889 |
| ABCB11/LRP2 | Sulfonylureas/Insulin | rs17539351 | 2 | 169766560 | T | C | -0.084 | 0.003 | 8.49E-134 | 606.0 | HbA1c | 389889 |
| ABCB11/LRP2 | Sulfonylureas/Insulin | rs2740590 | 2 | 169758316 | A | G | 0.071 | 0.003 | 4.22E-147 | 667.1 | HbA1c | 389889 |
| ABCB11/LRP2 | Sulfonylureas/Insulin | rs372482560 | 2 | 169790381 | T | C | 0.065 | 0.009 | 1.75E-13 | 54.3 | HbA1c | 389889 |
| ABCB11/LRP2 | Sulfonylureas/Insulin | rs3821120 | 2 | 169816936 | C | G | 0.032 | 0.002 | 5.27E-52 | 230.2 | HbA1c | 389889 |
| ABCB11/LRP2 | Sulfonylureas/Insulin | rs3843328 | 2 | 169656995 | A | G | -0.024 | 0.002 | 1.02E-25 | 109.9 | HbA1c | 389889 |
| ABCB11/LRP2 | Sulfonylureas/Insulin | rs4109426 | 2 | 169650906 | C | G | 0.016 | 0.002 | 1.68E-15 | 63.4 | HbA1c | 389889 |
| ABCB11/LRP2 | Sulfonylureas/Insulin | rs4668103 | 2 | 169603048 | G | A | 0.027 | 0.002 | 1.49E-31 | 136.6 | HbA1c | 389889 |
| ABCB11/LRP2 | Sulfonylureas/Insulin | rs491443 | 2 | 169769027 | C | A | 0.026 | 0.002 | 7.05E-39 | 170.1 | HbA1c | 389889 |
| ABCB11/LRP2 | Sulfonylureas/Insulin | rs532779 | 2 | 169703751 | A | T | -0.017 | 0.002 | 3.15E-14 | 57.6 | HbA1c | 389889 |
| ABCB11/LRP2 | Sulfonylureas/Insulin | rs56100844 | 2 | 169786707 | G | T | -0.132 | 0.009 | 1.34E-48 | 214.6 | HbA1c | 389889 |
| ABCB11/LRP2 | Sulfonylureas/Insulin | rs58431993 | 2 | 169625035 | A | G | 0.038 | 0.005 | 9.96E-15 | 59.9 | HbA1c | 389889 |
| ABCB11/LRP2 | Sulfonylureas/Insulin | rs62171055 | 2 | 169906208 | A | T | -0.037 | 0.006 | 4.68E-09 | 34.3 | HbA1c | 389889 |
| ABCB11/LRP2 | Sulfonylureas/Insulin | rs62174445 | 2 | 169588284 | G | T | -0.033 | 0.003 | 2.14E-24 | 103.9 | HbA1c | 389889 |
| ABCB11/LRP2 | Sulfonylureas/Insulin | rs72884870 | 2 | 169773338 | G | T | 0.075 | 0.007 | 4.91E-30 | 129.6 | HbA1c | 389889 |
| ABCB11/LRP2 | Sulfonylureas/Insulin | rs73032206 | 2 | 169693537 | G | A | 0.062 | 0.006 | 2.59E-27 | 117.2 | HbA1c | 389889 |
| ABCB11/LRP2 | Sulfonylureas/Insulin | rs73970001 | 2 | 169752890 | T | G | 0.078 | 0.009 | 6.87E-19 | 78.8 | HbA1c | 389889 |
| ABCB11/LRP2 | Sulfonylureas/Insulin | rs74648148 | 2 | 169774784 | C | G | 0.069 | 0.005 | 2.22E-47 | 209.0 | HbA1c | 389889 |
| ABCB11/LRP2 | Sulfonylureas/Insulin | rs75739013 | 2 | 169762590 | C | A | -0.075 | 0.006 | 5.21E-36 | 157.0 | HbA1c | 389889 |
| ABCB11/LRP2 | Sulfonylureas/Insulin | rs76031050 | 2 | 169816670 | A | G | -0.032 | 0.005 | 3.60E-10 | 39.3 | HbA1c | 389889 |
| ABCB11/LRP2 | Sulfonylureas/Insulin | rs77259705 | 2 | 169691051 | C | A | 0.016 | 0.003 | 1.36E-08 | 32.2 | HbA1c | 389889 |
| ABCB11/LRP2 | Sulfonylureas/Insulin | rs77501730 | 2 | 169702698 | T | A | 0.042 | 0.004 | 6.37E-21 | 88.1 | HbA1c | 389889 |
| ABCB11/LRP2 | Sulfonylureas/Insulin | rs77609521 | 2 | 169591521 | A | G | -0.053 | 0.004 | 9.72E-43 | 187.8 | HbA1c | 389889 |
| ABCB11/LRP2 | Sulfonylureas/Insulin | rs853770 | 2 | 169749841 | C | T | -0.047 | 0.002 | 4.29E-99 | 446.4 | HbA1c | 389889 |
| ABCB11/LRP2 | Sulfonylureas/Insulin | rs853773 | 2 | 169814347 | G | A | 0.070 | 0.002 | 1.00E-200 | 1163.8 | HbA1c | 389889 |
| ABCC8/KCNJ11 | Sulfonylureas | rs2214286 | 11 | 17376064 | C | T | -0.012 | 0.002 | 4.90E-08 | 29.8 | HbA1c | 389889 |
| ABCC8/KCNJ11 | Sulfonylureas | rs61880293 | 11 | 17376498 | C | T | 0.021 | 0.004 | 4.35E-08 | 30.0 | HbA1c | 389889 |
| ABCC8/KCNJ11 | Sulfonylureas | rs757110 | 11 | 17418477 | A | C | -0.022 | 0.002 | 1.57E-25 | 109.1 | HbA1c | 389889 |
| ABCC9/KCNJ8 | Sulfonylureas | rs10841891 | 12 | 21878493 | G | T | -0.015 | 0.002 | 6.82E-10 | 38.1 | HbA1c | 389889 |
| CPT1A | Sulfonylureas | rs188584129 | 11 | 68980762 | A | G | -0.043 | 0.008 | 3.89E-08 | 30.2 | HbA1c | 389889 |
| CPT1A | Sulfonylureas | rs61167756 | 11 | 68950835 | T | C | -0.021 | 0.003 | 1.76E-09 | 36.2 | HbA1c | 389889 |
| GANC | AGI | rs28401754 | 15 | 42116903 | C | T | 0.014 | 0.002 | 1.37E-11 | 45.7 | HbA1c | 389889 |
| GLP1R | GLP-1RA | rs10305518 | 6 | 39055012 | G | T | 0.028 | 0.004 | 2.32E-10 | 40.2 | HbA1c | 389889 |
| GLP1R | GLP-1RA | rs6904583 | 6 | 39358111 | T | C | 0.037 | 0.006 | 8.02E-09 | 33.3 | HbA1c | 389889 |
| GLP1R | GLP-1RA | rs910166 | 6 | 39033168 | A | G | 0.022 | 0.004 | 9.50E-10 | 37.4 | HbA1c | 389889 |
| GLP1R | GLP-1RA | rs9470964 | 6 | 39016096 | G | A | 0.027 | 0.005 | 8.12E-09 | 33.2 | HbA1c | 389889 |
| GPD1 | Metformin | rs111838971 | 12 | 50950883 | T | G | 0.025 | 0.004 | 5.14E-10 | 38.6 | HbA1c | 389889 |
| GPD1 | Metformin | rs61927768 | 12 | 50898728 | A | G | 0.021 | 0.002 | 7.17E-23 | 96.9 | HbA1c | 389889 |
| GPD1 | Metformin | rs836181 | 12 | 50484603 | T | G | -0.018 | 0.003 | 9.43E-09 | 33.0 | HbA1c | 389889 |
| INS | Sulfonylureas | rs11042596 | 11 | 2118860 | G | T | 0.014 | 0.002 | 2.70E-10 | 39.9 | HbA1c | 389889 |
| INS | Sulfonylureas | rs11564713 | 11 | 2191709 | T | C | -0.014 | 0.002 | 8.62E-11 | 42.1 | HbA1c | 389889 |
| INS | Sulfonylureas | rs11564725 | 11 | 2177369 | T | C | 0.025 | 0.002 | 7.60E-26 | 110.5 | HbA1c | 389889 |
| INS | Sulfonylureas | rs3213223 | 11 | 2156930 | A | G | 0.019 | 0.002 | 2.26E-15 | 62.8 | HbA1c | 389889 |
| KCNJ1 | Sulfonylureas | rs8705 | 11 | 128328913 | A | G | -0.014 | 0.002 | 3.76E-11 | 43.7 | HbA1c | 389889 |
| PPARG | TZDs | rs112496224 | 3 | 12301915 | T | C | -0.043 | 0.005 | 2.29E-16 | 67.3 | HbA1c | 389889 |
| PPARG | TZDs | rs1152002 | 3 | 12471871 | T | C | -0.015 | 0.002 | 9.62E-13 | 50.9 | HbA1c | 389889 |
| PPARG | TZDs | rs115633989 | 3 | 12176274 | G | A | -0.033 | 0.005 | 4.24E-13 | 52.5 | HbA1c | 389889 |
| PPARG | TZDs | rs116354045 | 3 | 11979131 | G | C | -0.039 | 0.007 | 1.04E-08 | 32.8 | HbA1c | 389889 |
| PPARG | TZDs | rs12492608 | 3 | 12605247 | T | A | -0.015 | 0.002 | 4.72E-09 | 34.3 | HbA1c | 389889 |
| PPARG | TZDs | rs13320580 | 3 | 11956181 | A | G | -0.013 | 0.002 | 2.48E-09 | 35.6 | HbA1c | 389889 |
| PPARG | TZDs | rs17029006 | 3 | 12329452 | T | C | 0.014 | 0.002 | 1.75E-09 | 36.2 | HbA1c | 389889 |
| PPARG | TZDs | rs17669026 | 3 | 12042835 | G | A | -0.015 | 0.003 | 1.19E-08 | 32.5 | HbA1c | 389889 |
| PPARG | TZDs | rs17669062 | 3 | 12043966 | C | T | -0.026 | 0.004 | 3.61E-12 | 48.3 | HbA1c | 389889 |
| PPARG | TZDs | rs17671592 | 3 | 12273414 | C | T | -0.027 | 0.005 | 3.19E-08 | 30.6 | HbA1c | 389889 |
| PPARG | TZDs | rs17819602 | 3 | 12507119 | G | A | -0.024 | 0.004 | 2.54E-10 | 40.0 | HbA1c | 389889 |
| PPARG | TZDs | rs1822534 | 3 | 12266804 | G | A | -0.041 | 0.002 | 1.16E-86 | 389.3 | HbA1c | 389889 |
| PPARG | TZDs | rs2055740 | 3 | 12791009 | A | G | -0.025 | 0.005 | 4.74E-08 | 29.8 | HbA1c | 389889 |
| PPARG | TZDs | rs2305398 | 3 | 12856856 | G | A | -0.015 | 0.002 | 1.28E-13 | 54.9 | HbA1c | 389889 |
| PPARG | TZDs | rs2600258 | 3 | 12004940 | G | A | -0.014 | 0.002 | 7.78E-09 | 33.3 | HbA1c | 389889 |
| PPARG | TZDs | rs2920499 | 3 | 12319058 | G | A | 0.017 | 0.002 | 1.12E-17 | 73.3 | HbA1c | 389889 |
| PPARG | TZDs | rs2921186 | 3 | 12385469 | T | C | 0.026 | 0.003 | 2.61E-19 | 80.7 | HbA1c | 389889 |
| PPARG | TZDs | rs307586 | 3 | 12110247 | T | C | -0.029 | 0.003 | 9.11E-20 | 82.8 | HbA1c | 389889 |
| PPARG | TZDs | rs3105363 | 3 | 12471070 | G | A | 0.026 | 0.002 | 1.84E-26 | 113.3 | HbA1c | 389889 |
| PPARG | TZDs | rs568984015 | 3 | 12371802 | T | A | 0.033 | 0.004 | 2.79E-20 | 85.1 | HbA1c | 389889 |
| PPARG | TZDs | rs62242087 | 3 | 12302208 | T | C | 0.025 | 0.004 | 6.54E-11 | 42.7 | HbA1c | 389889 |
| PPARG | TZDs | rs66603943 | 3 | 12484681 | C | T | -0.019 | 0.003 | 1.75E-13 | 54.3 | HbA1c | 389889 |
| PPARG | TZDs | rs6770851 | 3 | 12797563 | T | C | -0.022 | 0.003 | 1.02E-14 | 59.9 | HbA1c | 389889 |
| PPARG | TZDs | rs709159 | 3 | 12481203 | A | C | -0.019 | 0.002 | 9.47E-16 | 64.5 | HbA1c | 389889 |
| PPARG | TZDs | rs713178 | 3 | 12615984 | C | T | -0.014 | 0.002 | 8.45E-10 | 37.7 | HbA1c | 389889 |
| PPARG | TZDs | rs73130305 | 3 | 12621075 | T | C | -0.025 | 0.002 | 1.45E-25 | 109.2 | HbA1c | 389889 |
| PPARG | TZDs | rs75926229 | 3 | 12711971 | C | A | -0.015 | 0.002 | 7.52E-12 | 46.9 | HbA1c | 389889 |
| PPARG | TZDs | rs7631080 | 3 | 11977950 | A | T | -0.013 | 0.002 | 3.96E-08 | 30.2 | HbA1c | 389889 |
| PPARG | TZDs | rs78512510 | 3 | 12226789 | A | G | -0.033 | 0.005 | 4.92E-10 | 38.7 | HbA1c | 389889 |
| PPARG | TZDs | rs9872031 | 3 | 12496461 | A | G | 0.024 | 0.002 | 1.19E-31 | 137.0 | HbA1c | 389889 |
| RXRB | TZDs | rs112350825 | 6 | 32699144 | C | G | 0.017 | 0.002 | 9.17E-15 | 60.1 | HbA1c | 389889 |
| RXRB | TZDs | rs117821 | 6 | 32798711 | C | T | 0.012 | 0.002 | 2.20E-08 | 31.3 | HbA1c | 389889 |
| RXRB | TZDs | rs139345337 | 6 | 32790641 | C | T | -0.039 | 0.006 | 9.72E-12 | 46.4 | HbA1c | 389889 |
| RXRB | TZDs | rs200185390 | 6 | 32726605 | C | A | 0.036 | 0.003 | 9.07E-26 | 110.2 | HbA1c | 389889 |
| RXRB | TZDs | rs2294478 | 6 | 33098966 | A | C | -0.012 | 0.002 | 6.18E-09 | 33.8 | HbA1c | 389889 |
| RXRB | TZDs | rs2395380 | 6 | 32946837 | T | A | 0.028 | 0.003 | 3.66E-21 | 89.2 | HbA1c | 389889 |
| RXRB | TZDs | rs241424 | 6 | 32804934 | A | G | 0.023 | 0.002 | 5.87E-30 | 129.3 | HbA1c | 389889 |
| RXRB | TZDs | rs241426 | 6 | 32804553 | A | T | 0.016 | 0.002 | 5.98E-15 | 60.9 | HbA1c | 389889 |
| RXRB | TZDs | rs2621408 | 6 | 32747542 | G | C | 0.013 | 0.002 | 3.91E-10 | 39.2 | HbA1c | 389889 |
| RXRB | TZDs | rs2621417 | 6 | 32853607 | A | G | 0.021 | 0.002 | 3.19E-24 | 103.1 | HbA1c | 389889 |
| RXRB | TZDs | rs3104411 | 6 | 32683653 | A | G | 0.026 | 0.002 | 4.59E-34 | 148.1 | HbA1c | 389889 |
| RXRB | TZDs | rs3132135 | 6 | 32888861 | T | A | 0.021 | 0.003 | 3.65E-11 | 43.8 | HbA1c | 389889 |
| RXRB | TZDs | rs3132136 | 6 | 32853987 | A | G | 0.020 | 0.002 | 4.46E-16 | 66.0 | HbA1c | 389889 |
| RXRB | TZDs | rs372546769 | 6 | 32966860 | G | A | 0.013 | 0.002 | 3.51E-09 | 34.9 | HbA1c | 389889 |
| RXRB | TZDs | rs3892710 | 6 | 32682862 | T | C | 0.016 | 0.003 | 1.52E-08 | 32.0 | HbA1c | 389889 |
| RXRB | TZDs | rs564030769 | 6 | 33634085 | A | T | 0.029 | 0.005 | 4.50E-08 | 29.9 | HbA1c | 389889 |
| RXRB | TZDs | rs568102960 | 6 | 32683287 | G | A | 0.018 | 0.003 | 2.71E-08 | 30.9 | HbA1c | 389889 |
| RXRB | TZDs | rs62397687 | 6 | 32802009 | T | C | 0.032 | 0.003 | 1.69E-34 | 150.1 | HbA1c | 389889 |
| RXRB | TZDs | rs7383287 | 6 | 32783086 | G | A | 0.024 | 0.002 | 2.62E-22 | 94.4 | HbA1c | 389889 |
| RXRB | TZDs | rs7760769 | 6 | 32746436 | A | G | 0.019 | 0.002 | 5.27E-22 | 93.0 | HbA1c | 389889 |
| RXRB | TZDs | rs7764819 | 6 | 32680576 | G | T | -0.019 | 0.003 | 4.83E-10 | 38.7 | HbA1c | 389889 |
| RXRB | TZDs | rs9275601 | 6 | 32682664 | T | C | 0.017 | 0.002 | 5.84E-17 | 70.0 | HbA1c | 389889 |
| RXRB | TZDs | rs9276084 | 6 | 32696666 | A | C | 0.035 | 0.003 | 5.04E-33 | 143.3 | HbA1c | 389889 |
| RXRB | TZDs | rs9276410 | 6 | 32710396 | A | G | 0.016 | 0.002 | 4.15E-14 | 57.1 | HbA1c | 389889 |
| SLC5A1 | SGLT2i | rs12484925 | 22 | 32411660 | T | C | -0.020 | 0.003 | 4.71E-12 | 47.8 | HbA1c | 389889 |
| SLC5A1 | SGLT2i | rs136420 | 22 | 32576071 | A | G | -0.014 | 0.002 | 2.11E-09 | 35.9 | HbA1c | 389889 |
| SLC5A1 | SGLT2i | rs3986040 | 22 | 32553177 | C | T | -0.029 | 0.003 | 3.35E-17 | 71.1 | HbA1c | 389889 |
| SLC5A1 | SGLT2i | rs5754109 | 22 | 32872593 | A | G | 0.015 | 0.002 | 1.85E-11 | 45.1 | HbA1c | 389889 |
| SLC5A1 | SGLT2i | rs60820868 | 22 | 32580130 | A | T | -0.017 | 0.003 | 2.35E-09 | 35.7 | HbA1c | 389889 |
| SLC5A1 | SGLT2i | rs75009211 | 22 | 32347308 | C | T | 0.019 | 0.003 | 3.15E-09 | 35.1 | HbA1c | 389889 |
| SLC5A1 | SGLT2i | rs9606885 | 22 | 32334943 | G | A | -0.014 | 0.002 | 3.26E-12 | 48.5 | HbA1c | 389889 |
| SLC5A1 | SGLT2i | rs9606898 | 22 | 32445218 | C | A | -0.024 | 0.003 | 2.11E-19 | 81.1 | HbA1c | 389889 |
| SLC5A2 | SGLT2i | rs1232538 | 16 | 31833812 | T | G | 0.014 | 0.002 | 1.19E-09 | 37.0 | HbA1c | 389889 |
| SLC5A2 | SGLT2i | rs12929023 | 16 | 31380464 | A | G | 0.016 | 0.003 | 1.54E-08 | 32.0 | HbA1c | 389889 |
| SLC5A2 | SGLT2i | rs12932429 | 16 | 31403145 | C | T | -0.028 | 0.005 | 1.56E-09 | 36.5 | HbA1c | 389889 |
| SLC5A2 | SGLT2i | rs17855121 | 16 | 31004169 | C | T | 0.015 | 0.002 | 4.00E-11 | 43.6 | HbA1c | 389889 |
| SLC5A2 | SGLT2i | rs28675289 | 16 | 31463252 | T | C | -0.038 | 0.005 | 6.75E-15 | 60.7 | HbA1c | 389889 |
| SLC5A2 | SGLT2i | rs28692853 | 16 | 31573030 | A | C | -0.014 | 0.002 | 2.60E-12 | 49.0 | HbA1c | 389889 |
| SLC5A2 | SGLT2i | rs45625038 | 16 | 31418975 | T | C | 0.039 | 0.006 | 2.99E-11 | 44.2 | HbA1c | 389889 |
| SLC5A2 | SGLT2i | rs8050500 | 16 | 31404571 | C | T | -0.026 | 0.002 | 2.90E-38 | 167.3 | HbA1c | 389889 |
| VEGFA/SLC29A1 | Sulfonylureas/TZDs | rs10434 | 6 | 43753212 | G | A | 0.011 | 0.002 | 2.86E-08 | 30.8 | HbA1c | 389889 |
| VEGFA/SLC29A1 | Sulfonylureas/TZDs | rs1570360 | 6 | 43737830 | G | A | 0.018 | 0.002 | 8.23E-17 | 69.4 | HbA1c | 389889 |
| VEGFA/SLC29A1 | Sulfonylureas/TZDs | rs2894536 | 6 | 43801878 | T | C | -0.016 | 0.003 | 3.63E-09 | 34.8 | HbA1c | 389889 |
| VEGFA/SLC29A1 | Sulfonylureas/TZDs | rs9394963 | 6 | 43719391 | T | G | -0.012 | 0.002 | 1.65E-08 | 31.9 | HbA1c | 389889 |
| VEGFA/SLC29A1 | Sulfonylureas/TZDs | rs943070 | 6 | 43740451 | G | C | 0.017 | 0.003 | 2.62E-11 | 44.4 | HbA1c | 389889 |
| VEGFA/SLC29A1 | Sulfonylureas/TZDs | rs9472125 | 6 | 43756169 | T | C | -0.030 | 0.003 | 4.26E-19 | 79.7 | HbA1c | 389889 |
| VEGFA/SLC29A1 | Sulfonylureas/TZDs | rs998584 | 6 | 43757896 | A | C | 0.022 | 0.002 | 2.00E-27 | 117.7 | HbA1c | 389889 |

| **Table S4. Detailed information of positive control analysis** | | | | | | | | | | |
| --- | --- | --- | --- | --- | --- | --- | --- | --- | --- | --- |
| **Exposure** | **Outcome** | **Method** | **nSNP** | **beta** | **se** | **pval** | **OR** | **OR_lci95** | **OR_uci95** | **P_FDR** |
| ABCB11 | random blood glocuse | Inverse variance weighted | 2 | -0.201 | 0.109 | 0.066 | 0.818 | 0.660 | 1.014 | 0.081 |
| ABCB11 | T2DM | Wald ratio | 1 | -0.186 | 0.149 | 0.212 | 0.830 | 0.620 | 1.112 | 0.238 |
| ABCB11/LRP2 | T2DM | Inverse variance weighted | 9 | 0.055 | 0.020 | 0.007 | 1.056 | 1.015 | 1.099 | 0.012 |
| ABCB11/LRP2 | T2DM | MR Egger | 9 | 0.017 | 0.057 | 0.768 | 1.018 | 0.911 | 1.137 | 0.907 |
| ABCB11/LRP2 | T2DM | Weighted median | 9 | 0.043 | 0.024 | 0.073 | 1.044 | 0.996 | 1.095 | 0.079 |
| ABCC8/KCNJ11 | random blood glocuse | Inverse variance weighted | 2 | -0.643 | 0.147 | 0.000 | 0.525 | 0.394 | 0.701 | 0.000 |
| ABCC8/KCNJ11 | T2DM | Wald ratio | 1 | -0.591 | 0.171 | 0.001 | 0.554 | 0.396 | 0.775 | 0.001 |
| ABCC9/KCNJ8 | random blood glocuse | Wald ratio | 1 | -0.092 | 0.184 | 0.617 | 0.912 | 0.636 | 1.308 | 0.640 |
| ABCC9/KCNJ8 | T2DM | Wald ratio | 1 | -0.404 | 0.150 | 0.007 | 0.667 | 0.497 | 0.896 | 0.012 |
| CPT1A | random blood glocuse | Inverse variance weighted | 2 | -0.261 | 0.139 | 0.061 | 0.770 | 0.586 | 1.013 | 0.078 |
| CPT1A | T2DM | Wald ratio | 1 | -0.465 | 0.155 | 0.003 | 0.628 | 0.464 | 0.851 | 0.005 |
| GANC | random blood glocuse | Wald ratio | 1 | -0.675 | 0.168 | 0.000 | 0.509 | 0.366 | 0.709 | 0.000 |
| GANC | T2DM | Wald ratio | 1 | -0.535 | 0.137 | 0.000 | 0.586 | 0.447 | 0.767 | 0.000 |
| GLP1R | random blood glocuse | Inverse variance weighted | 3 | -0.576 | 0.117 | 0.000 | 0.562 | 0.447 | 0.707 | 0.000 |
| GLP1R | T2DM | Inverse variance weighted | 4 | -0.347 | 0.077 | 0.000 | 0.707 | 0.607 | 0.822 | 0.000 |
| GLP1R | random blood glocuse | MR Egger | 3 | 0.708 | 0.861 | 0.562 | 2.030 | 0.376 | 10.972 | 0.811 |
| GLP1R | T2DM | MR Egger | 4 | 0.017 | 0.456 | 0.974 | 1.017 | 0.416 | 2.485 | 0.974 |
| GLP1R | random blood glocuse | Weighted median | 3 | -0.631 | 0.158 | 0.000 | 0.532 | 0.391 | 0.725 | 0.000 |
| GLP1R | T2DM | Weighted median | 4 | -0.299 | 0.100 | 0.003 | 0.741 | 0.609 | 0.903 | 0.005 |
| GPD1 | random blood glocuse | Inverse variance weighted | 3 | 0.174 | 0.088 | 0.047 | 1.190 | 1.002 | 1.413 | 0.063 |
| GPD1 | T2DM | Wald ratio | 1 | -0.202 | 0.095 | 0.032 | 0.817 | 0.679 | 0.983 | 0.047 |
| GPD1 | random blood glocuse | MR Egger | 3 | 1.232 | 0.881 | 0.395 | 3.429 | 0.610 | 19.281 | 0.811 |
| GPD1 | random blood glocuse | Weighted median | 3 | 0.178 | 0.099 | 0.072 | 1.195 | 0.984 | 1.452 | 0.079 |
| INS | random blood glocuse | Inverse variance weighted | 3 | -0.271 | 0.168 | 0.107 | 0.762 | 0.548 | 1.060 | 0.125 |
| INS | random blood glocuse | MR Egger | 3 | -1.578 | 0.763 | 0.287 | 0.206 | 0.046 | 0.920 | 0.811 |
| INS | random blood glocuse | Weighted median | 3 | -0.283 | 0.138 | 0.041 | 0.754 | 0.575 | 0.989 | 0.056 |
| KCNJ1 | random blood glocuse | Wald ratio | 1 | -0.063 | 0.172 | 0.712 | 0.939 | 0.670 | 1.314 | 0.712 |
| KCNJ1 | T2DM | Wald ratio | 1 | -0.128 | 0.141 | 0.365 | 0.880 | 0.668 | 1.160 | 0.393 |
| PPARG | random blood glocuse | Inverse variance weighted | 26 | -0.115 | 0.030 | 0.000 | 0.891 | 0.841 | 0.945 | 0.000 |
| PPARG | T2DM | Inverse variance weighted | 11 | -0.170 | 0.052 | 0.001 | 0.844 | 0.762 | 0.934 | 0.002 |
| PPARG | random blood glocuse | MR Egger | 26 | -0.064 | 0.087 | 0.464 | 0.938 | 0.791 | 1.111 | 0.811 |
| PPARG | T2DM | MR Egger | 11 | -0.133 | 0.161 | 0.429 | 0.875 | 0.639 | 1.200 | 0.811 |
| PPARG | random blood glocuse | Weighted median | 26 | -0.085 | 0.039 | 0.029 | 0.919 | 0.852 | 0.991 | 0.047 |
| PPARG | T2DM | Weighted median | 11 | -0.135 | 0.041 | 0.001 | 0.874 | 0.806 | 0.948 | 0.003 |
| RXRB | random blood glocuse | Inverse variance weighted | 18 | -0.276 | 0.030 | 0.000 | 0.759 | 0.715 | 0.805 | 0.000 |
| RXRB | T2DM | Inverse variance weighted | 3 | -0.229 | 0.057 | 0.000 | 0.795 | 0.711 | 0.890 | 0.000 |
| RXRB | random blood glocuse | MR Egger | 18 | -0.212 | 0.095 | 0.039 | 0.809 | 0.672 | 0.973 | 0.511 |
| RXRB | T2DM | MR Egger | 3 | -0.254 | 0.279 | 0.529 | 0.775 | 0.449 | 1.339 | 0.811 |
| RXRB | random blood glocuse | Weighted median | 18 | -0.305 | 0.042 | 0.000 | 0.737 | 0.678 | 0.801 | 0.000 |
| RXRB | T2DM | Weighted median | 3 | -0.213 | 0.066 | 0.001 | 0.808 | 0.710 | 0.919 | 0.003 |
| SLC5A1 | random blood glocuse | Inverse variance weighted | 7 | -0.380 | 0.060 | 0.000 | 0.684 | 0.609 | 0.769 | 0.000 |
| SLC5A1 | T2DM | Inverse variance weighted | 3 | -0.298 | 0.083 | 0.000 | 0.742 | 0.631 | 0.873 | 0.001 |
| SLC5A1 | random blood glocuse | MR Egger | 7 | -0.385 | 0.234 | 0.161 | 0.681 | 0.430 | 1.077 | 0.811 |
| SLC5A1 | T2DM | MR Egger | 3 | -0.927 | 0.637 | 0.383 | 0.396 | 0.114 | 1.380 | 0.811 |
| SLC5A1 | random blood glocuse | Weighted median | 7 | -0.368 | 0.080 | 0.000 | 0.692 | 0.591 | 0.810 | 0.000 |
| SLC5A1 | T2DM | Weighted median | 3 | -0.345 | 0.106 | 0.001 | 0.708 | 0.576 | 0.872 | 0.003 |
| SLC5A2 | random blood glocuse | Inverse variance weighted | 8 | -0.201 | 0.052 | 0.000 | 0.818 | 0.738 | 0.906 | 0.000 |
| SLC5A2 | T2DM | Inverse variance weighted | 2 | -0.336 | 0.124 | 0.007 | 0.715 | 0.560 | 0.912 | 0.012 |
| SLC5A2 | random blood glocuse | MR Egger | 8 | 0.070 | 0.149 | 0.654 | 1.073 | 0.801 | 1.436 | 0.851 |
| SLC5A2 | random blood glocuse | Weighted median | 8 | -0.148 | 0.073 | 0.043 | 0.863 | 0.748 | 0.995 | 0.056 |
| VEGFA/SLC29A1 | random blood glocuse | Inverse variance weighted | 5 | -0.133 | 0.063 | 0.034 | 0.875 | 0.774 | 0.990 | 0.047 |
| VEGFA/SLC29A1 | T2DM | Inverse variance weighted | 2 | -0.424 | 0.182 | 0.020 | 0.655 | 0.458 | 0.936 | 0.031 |
| VEGFA/SLC29A1 | random blood glocuse | MR Egger | 5 | -0.022 | 0.195 | 0.917 | 0.978 | 0.668 | 1.433 | 0.974 |
| VEGFA/SLC29A1 | random blood glocuse | Weighted median | 5 | -0.123 | 0.076 | 0.104 | 0.884 | 0.762 | 1.026 | 0.104 |

| **Table S5. Detailed information of all results in two-sample MR analysis and effect scaled by random blood glucose** | | | | | | | | | | | | |
| --- | --- | --- | --- | --- | --- | --- | --- | --- | --- | --- | --- | --- |
| **Exposure** | **Outcome** | **Method** | **nSNP** | **beta** | **beta_scaled** | **se** | **pval** | **OR** | **OR_scaled** | **OR_lci95** | **OR_uci95** | **P_FDR** |
| RXRB | AF | Inverse variance weighted | 14 | -0.132 | -0.246 | 0.037 | 3.20E-04 | 0.877 | 0.782 | 0.816 | 0.942 | 0.017 |
| ABCC8/KCNJ11 | PT | Inverse variance weighted | 3 | -0.364 | -0.681 | 0.108 | 7.39E-04 | 0.695 | 0.506 | 0.562 | 0.858 | 0.020 |
| SLC5A2 | RBBB | Inverse variance weighted | 8 | -0.164 | -0.306 | 0.051 | 0.001 | 0.849 | 0.736 | 0.768 | 0.938 | 0.024 |
| GPD1 | RBBB | Inverse variance weighted | 2 | 0.280 | 0.524 | 0.097 | 0.004 | 1.323 | 1.688 | 1.094 | 1.601 | 0.053 |
| RXRB | AVB | Inverse variance weighted | 14 | 0.093 | 0.174 | 0.033 | 0.005 | 1.098 | 1.190 | 1.028 | 1.172 | 0.055 |
| SLC5A2 | LBBB | Inverse variance weighted | 8 | 0.134 | 0.250 | 0.051 | 0.009 | 1.143 | 1.283 | 1.034 | 1.263 | 0.080 |
| ABCC8/KCNJ11 | PT | Weighted median | 3 | -0.319 | -0.596 | 0.119 | 0.007 | 0.727 | 0.551 | 0.576 | 0.918 | 0.131 |
| RXRB | AF | Weighted median | 14 | -0.132 | -0.246 | 0.048 | 0.006 | 0.877 | 0.782 | 0.798 | 0.963 | 0.131 |
| VEGFA/SLC29A1 | AF | Inverse variance weighted | 6 | 0.142 | 0.265 | 0.060 | 0.019 | 1.153 | 1.304 | 1.024 | 1.297 | 0.145 |
| ABCC9/KCNJ8 | AVB | Wald ratio | 1 | -0.348 | -0.651 | 0.164 | 0.034 | 0.706 | 0.522 | 0.512 | 0.973 | 0.184 |
| PPARG | PT | Inverse variance weighted | 26 | 0.067 | 0.126 | 0.032 | 0.034 | 1.070 | 1.134 | 1.005 | 1.138 | 0.184 |
| VEGFA/SLC29A1 | PT | Inverse variance weighted | 6 | 0.181 | 0.339 | 0.085 | 0.033 | 1.199 | 1.403 | 1.014 | 1.416 | 0.184 |
| PPARG | AF | Weighted median | 24 | 0.102 | 0.191 | 0.048 | 0.034 | 1.107 | 1.210 | 1.008 | 1.217 | 0.198 |
| SLC5A2 | RBBB | Weighted median | 8 | -0.142 | -0.265 | 0.066 | 0.032 | 0.868 | 0.767 | 0.762 | 0.988 | 0.198 |
| VEGFA/SLC29A1 | LBBB | Weighted median | 6 | 0.159 | 0.297 | 0.074 | 0.031 | 1.172 | 1.345 | 1.014 | 1.354 | 0.198 |
| VEGFA/SLC29A1 | PT | Weighted median | 6 | 0.201 | 0.376 | 0.087 | 0.021 | 1.223 | 1.456 | 1.030 | 1.451 | 0.198 |
| RXRB | AVB | Weighted median | 14 | 0.087 | 0.163 | 0.042 | 0.041 | 1.091 | 1.176 | 1.004 | 1.186 | 0.203 |
| RXRB | PT | Inverse variance weighted | 14 | 0.077 | 0.143 | 0.040 | 0.055 | 1.080 | 1.154 | 0.998 | 1.167 | 0.271 |
| SLC5A2 | LBBB | Weighted median | 8 | 0.124 | 0.231 | 0.068 | 0.067 | 1.132 | 1.260 | 0.991 | 1.292 | 0.293 |
| VEGFA/SLC29A1 | AVB | Weighted median | 6 | 0.120 | 0.224 | 0.070 | 0.085 | 1.127 | 1.251 | 0.984 | 1.293 | 0.331 |
| PPARG | AF | Inverse variance weighted | 24 | 0.087 | 0.163 | 0.050 | 0.079 | 1.091 | 1.177 | 0.990 | 1.202 | 0.355 |
| GANC | AVB | Wald ratio | 1 | -0.273 | -0.511 | 0.164 | 0.097 | 0.761 | 0.600 | 0.551 | 1.050 | 0.402 |
| VEGFA/SLC29A1 | AVB | Inverse variance weighted | 6 | 0.096 | 0.180 | 0.061 | 0.117 | 1.101 | 1.197 | 0.976 | 1.242 | 0.449 |
| ABCC8/KCNJ11 | LBBB | Inverse variance weighted | 3 | -0.147 | -0.275 | 0.101 | 0.145 | 0.863 | 0.760 | 0.709 | 1.052 | 0.472 |
| CPT1A | PT | Inverse variance weighted | 2 | -0.322 | -0.602 | 0.223 | 0.149 | 0.725 | 0.548 | 0.468 | 1.122 | 0.472 |
| GPD1 | AF | Inverse variance weighted | 2 | 0.155 | 0.289 | 0.106 | 0.145 | 1.167 | 1.335 | 0.948 | 1.437 | 0.472 |
| VEGFA/SLC29A1 | LBBB | Inverse variance weighted | 6 | 0.121 | 0.227 | 0.086 | 0.158 | 1.129 | 1.254 | 0.954 | 1.336 | 0.475 |
| ABCC9/KCNJ8 | AF | Wald ratio | 1 | 0.238 | 0.445 | 0.180 | 0.186 | 1.269 | 1.561 | 0.891 | 1.807 | 0.480 |
| ABCC9/KCNJ8 | LBBB | Wald ratio | 1 | 0.221 | 0.413 | 0.165 | 0.181 | 1.247 | 1.511 | 0.902 | 1.723 | 0.480 |
| SLC5A1 | AVB | Inverse variance weighted | 6 | 0.090 | 0.169 | 0.067 | 0.180 | 1.095 | 1.184 | 0.959 | 1.249 | 0.480 |
| GANC | PT | Wald ratio | 1 | -0.247 | -0.462 | 0.198 | 0.211 | 0.781 | 0.630 | 0.530 | 1.150 | 0.503 |
| GPD1 | AVB | Inverse variance weighted | 2 | 0.120 | 0.224 | 0.096 | 0.214 | 1.127 | 1.251 | 0.933 | 1.362 | 0.503 |
| ABCC8/KCNJ11 | RBBB | Weighted median | 3 | 0.149 | 0.278 | 0.105 | 0.155 | 1.161 | 1.321 | 0.945 | 1.425 | 0.529 |
| VEGFA/SLC29A1 | AF | Weighted median | 6 | 0.113 | 0.211 | 0.081 | 0.166 | 1.119 | 1.234 | 0.954 | 1.313 | 0.529 |
| GLP1R | RBBB | Weighted median | 3 | -0.175 | -0.326 | 0.132 | 0.187 | 0.840 | 0.722 | 0.648 | 1.088 | 0.545 |
| GPD1 | LBBB | Inverse variance weighted | 2 | -0.113 | -0.211 | 0.097 | 0.244 | 0.893 | 0.809 | 0.738 | 1.080 | 0.548 |
| GLP1R | RBBB | Inverse variance weighted | 3 | -0.119 | -0.223 | 0.110 | 0.278 | 0.888 | 0.800 | 0.716 | 1.101 | 0.601 |
| ABCC9/KCNJ8 | PT | Wald ratio | 1 | -0.202 | -0.378 | 0.197 | 0.304 | 0.817 | 0.685 | 0.556 | 1.201 | 0.612 |
| CPT1A | LBBB | Inverse variance weighted | 2 | -0.149 | -0.279 | 0.167 | 0.371 | 0.861 | 0.756 | 0.621 | 1.195 | 0.612 |
| GLP1R | AVB | Inverse variance weighted | 3 | 0.110 | 0.205 | 0.109 | 0.315 | 1.116 | 1.227 | 0.901 | 1.381 | 0.612 |
| GLP1R | LBBB | Inverse variance weighted | 3 | 0.101 | 0.189 | 0.110 | 0.357 | 1.106 | 1.208 | 0.892 | 1.372 | 0.612 |
| GLP1R | PT | Inverse variance weighted | 3 | 0.130 | 0.243 | 0.131 | 0.322 | 1.139 | 1.275 | 0.881 | 1.472 | 0.612 |
| RXRB | RBBB | Inverse variance weighted | 14 | -0.031 | -0.058 | 0.034 | 0.358 | 0.970 | 0.944 | 0.908 | 1.035 | 0.612 |
| SLC5A1 | RBBB | Inverse variance weighted | 6 | 0.082 | 0.153 | 0.092 | 0.374 | 1.085 | 1.165 | 0.906 | 1.299 | 0.612 |
| SLC5A2 | AF | Inverse variance weighted | 8 | 0.055 | 0.102 | 0.056 | 0.330 | 1.056 | 1.107 | 0.946 | 1.179 | 0.612 |
| ABCC8/KCNJ11 | RBBB | Inverse variance weighted | 3 | 0.121 | 0.226 | 0.144 | 0.403 | 1.128 | 1.253 | 0.850 | 1.498 | 0.622 |
| GANC | LBBB | Wald ratio | 1 | -0.141 | -0.264 | 0.166 | 0.395 | 0.869 | 0.768 | 0.628 | 1.202 | 0.622 |
| ABCC8/KCNJ11 | LBBB | Weighted median | 3 | -0.117 | -0.219 | 0.098 | 0.231 | 0.889 | 0.803 | 0.734 | 1.078 | 0.623 |
| RXRB | LBBB | Inverse variance weighted | 14 | 0.035 | 0.065 | 0.044 | 0.429 | 1.036 | 1.068 | 0.950 | 1.129 | 0.644 |
| VEGFA/SLC29A1 | RBBB | Inverse variance weighted | 6 | 0.041 | 0.077 | 0.055 | 0.458 | 1.042 | 1.080 | 0.935 | 1.162 | 0.669 |
| GLP1R | AVB | Weighted median | 3 | 0.115 | 0.215 | 0.128 | 0.368 | 1.122 | 1.240 | 0.873 | 1.442 | 0.680 |
| GLP1R | LBBB | Weighted median | 3 | 0.133 | 0.249 | 0.128 | 0.296 | 1.143 | 1.283 | 0.890 | 1.467 | 0.680 |
| GLP1R | PT | Weighted median | 3 | 0.160 | 0.298 | 0.147 | 0.278 | 1.173 | 1.348 | 0.879 | 1.565 | 0.680 |
| PPARG | PT | Weighted median | 26 | 0.045 | 0.084 | 0.046 | 0.330 | 1.046 | 1.088 | 0.955 | 1.145 | 0.680 |
| RXRB | PT | Weighted median | 14 | 0.048 | 0.089 | 0.053 | 0.369 | 1.049 | 1.093 | 0.945 | 1.164 | 0.680 |
| SLC5A1 | AVB | Weighted median | 6 | 0.074 | 0.138 | 0.081 | 0.362 | 1.077 | 1.148 | 0.919 | 1.262 | 0.680 |
| ABCC8/KCNJ11 | AF | Inverse variance weighted | 3 | -0.068 | -0.127 | 0.099 | 0.493 | 0.934 | 0.881 | 0.770 | 1.134 | 0.701 |
| GLP1R | AF | Inverse variance weighted | 3 | 0.080 | 0.149 | 0.120 | 0.508 | 1.083 | 1.160 | 0.856 | 1.370 | 0.703 |
| GLP1R | AF | Weighted median | 3 | 0.106 | 0.199 | 0.139 | 0.446 | 1.112 | 1.220 | 0.846 | 1.462 | 0.715 |
| PPARG | RBBB | Weighted median | 26 | -0.028 | -0.052 | 0.039 | 0.469 | 0.972 | 0.949 | 0.902 | 1.049 | 0.715 |
| SLC5A1 | PT | Weighted median | 6 | 0.075 | 0.140 | 0.104 | 0.470 | 1.078 | 1.150 | 0.880 | 1.320 | 0.715 |
| SLC5A1 | RBBB | Weighted median | 6 | 0.078 | 0.146 | 0.096 | 0.416 | 1.082 | 1.158 | 0.895 | 1.306 | 0.715 |
| CPT1A | AVB | Inverse variance weighted | 2 | 0.177 | 0.331 | 0.285 | 0.535 | 1.193 | 1.392 | 0.683 | 2.086 | 0.719 |
| PPARG | AVB | Inverse variance weighted | 26 | -0.016 | -0.030 | 0.026 | 0.546 | 0.984 | 0.971 | 0.934 | 1.036 | 0.719 |
| GPD1 | PT | Inverse variance weighted | 2 | -0.062 | -0.116 | 0.116 | 0.592 | 0.940 | 0.890 | 0.749 | 1.179 | 0.743 |
| SLC5A1 | PT | Inverse variance weighted | 6 | 0.044 | 0.082 | 0.081 | 0.587 | 1.045 | 1.086 | 0.892 | 1.225 | 0.743 |
| CPT1A | AF | Inverse variance weighted | 2 | 0.085 | 0.159 | 0.182 | 0.639 | 1.089 | 1.173 | 0.763 | 1.555 | 0.748 |
| CPT1A | RBBB | Inverse variance weighted | 2 | -0.079 | -0.147 | 0.167 | 0.638 | 0.924 | 0.863 | 0.666 | 1.283 | 0.748 |
| PPARG | RBBB | Inverse variance weighted | 26 | -0.015 | -0.027 | 0.029 | 0.618 | 0.985 | 0.973 | 0.930 | 1.044 | 0.748 |
| SLC5A2 | AVB | Inverse variance weighted | 8 | -0.023 | -0.043 | 0.051 | 0.651 | 0.977 | 0.958 | 0.885 | 1.079 | 0.748 |
| SLC5A1 | AF | Inverse variance weighted | 6 | 0.027 | 0.050 | 0.074 | 0.716 | 1.027 | 1.052 | 0.888 | 1.188 | 0.794 |
| SLC5A2 | PT | Inverse variance weighted | 8 | -0.022 | -0.041 | 0.061 | 0.720 | 0.978 | 0.960 | 0.868 | 1.102 | 0.794 |
| ABCC9/KCNJ8 | RBBB | Wald ratio | 1 | 0.054 | 0.101 | 0.165 | 0.743 | 1.056 | 1.106 | 0.763 | 1.459 | 0.803 |
| PPARG | LBBB | Weighted median | 26 | -0.019 | -0.036 | 0.038 | 0.612 | 0.981 | 0.965 | 0.911 | 1.056 | 0.857 |
| SLC5A2 | AF | Weighted median | 8 | 0.039 | 0.073 | 0.075 | 0.601 | 1.040 | 1.076 | 0.898 | 1.204 | 0.857 |
| RXRB | RBBB | Weighted median | 14 | -0.017 | -0.032 | 0.043 | 0.692 | 0.983 | 0.969 | 0.904 | 1.069 | 0.889 |
| SLC5A2 | AVB | Weighted median | 8 | -0.027 | -0.050 | 0.061 | 0.661 | 0.974 | 0.951 | 0.864 | 1.097 | 0.889 |
| VEGFA/SLC29A1 | RBBB | Weighted median | 6 | 0.027 | 0.050 | 0.072 | 0.711 | 1.027 | 1.051 | 0.893 | 1.181 | 0.889 |
| ABCC8/KCNJ11 | AF | MR Egger | 3 | -0.157 | -0.293 | 0.488 | 0.802 | 0.855 | 0.746 | 0.329 | 2.223 | 0.893 |
| ABCC8/KCNJ11 | LBBB | MR Egger | 3 | 0.454 | 0.849 | 0.411 | 0.468 | 1.575 | 2.337 | 0.703 | 3.527 | 0.893 |
| ABCC8/KCNJ11 | PT | MR Egger | 3 | -0.516 | -0.965 | 0.630 | 0.563 | 0.597 | 0.381 | 0.174 | 2.051 | 0.893 |
| ABCC8/KCNJ11 | RBBB | MR Egger | 3 | -0.319 | -0.596 | 0.811 | 0.762 | 0.727 | 0.551 | 0.148 | 3.566 | 0.893 |
| GLP1R | AF | MR Egger | 3 | 0.460 | 0.859 | 0.561 | 0.563 | 1.583 | 2.361 | 0.527 | 4.755 | 0.893 |
| GLP1R | AVB | MR Egger | 3 | 0.151 | 0.283 | 0.509 | 0.816 | 1.163 | 1.327 | 0.429 | 3.155 | 0.893 |
| GLP1R | LBBB | MR Egger | 3 | 0.326 | 0.610 | 0.512 | 0.639 | 1.386 | 1.840 | 0.508 | 3.783 | 0.893 |
| GLP1R | PT | MR Egger | 3 | -0.364 | -0.680 | 0.612 | 0.659 | 0.695 | 0.507 | 0.209 | 2.308 | 0.893 |
| GLP1R | RBBB | MR Egger | 3 | -0.622 | -1.162 | 0.513 | 0.439 | 0.537 | 0.313 | 0.196 | 1.469 | 0.893 |
| PPARG | AF | MR Egger | 24 | -0.042 | -0.078 | 0.138 | 0.766 | 0.959 | 0.925 | 0.732 | 1.258 | 0.893 |
| PPARG | AVB | MR Egger | 26 | -0.023 | -0.043 | 0.073 | 0.758 | 0.977 | 0.958 | 0.847 | 1.128 | 0.893 |
| PPARG | LBBB | MR Egger | 26 | -0.055 | -0.103 | 0.074 | 0.460 | 0.946 | 0.902 | 0.819 | 1.093 | 0.893 |
| PPARG | PT | MR Egger | 26 | -0.042 | -0.078 | 0.088 | 0.637 | 0.959 | 0.925 | 0.808 | 1.139 | 0.893 |
| PPARG | RBBB | MR Egger | 26 | 0.059 | 0.110 | 0.081 | 0.476 | 1.061 | 1.116 | 0.904 | 1.244 | 0.893 |
| RXRB | AF | MR Egger | 14 | -0.142 | -0.265 | 0.121 | 0.262 | 0.868 | 0.767 | 0.685 | 1.099 | 0.893 |
| RXRB | AVB | MR Egger | 14 | 0.065 | 0.121 | 0.110 | 0.565 | 1.067 | 1.129 | 0.861 | 1.323 | 0.893 |
| RXRB | LBBB | MR Egger | 14 | 0.036 | 0.067 | 0.152 | 0.816 | 1.037 | 1.070 | 0.770 | 1.396 | 0.893 |
| SLC5A1 | AF | MR Egger | 6 | 0.181 | 0.339 | 0.306 | 0.585 | 1.199 | 1.403 | 0.658 | 2.183 | 0.893 |
| SLC5A1 | AVB | MR Egger | 6 | 0.183 | 0.342 | 0.278 | 0.547 | 1.201 | 1.408 | 0.696 | 2.072 | 0.893 |
| SLC5A1 | LBBB | MR Egger | 6 | 0.279 | 0.521 | 0.280 | 0.376 | 1.321 | 1.684 | 0.763 | 2.289 | 0.893 |
| SLC5A1 | PT | MR Egger | 6 | 0.407 | 0.761 | 0.334 | 0.290 | 1.502 | 2.140 | 0.781 | 2.890 | 0.893 |
| SLC5A1 | RBBB | MR Egger | 6 | 0.402 | 0.751 | 0.391 | 0.362 | 1.494 | 2.119 | 0.695 | 3.215 | 0.893 |
| SLC5A2 | AF | MR Egger | 8 | -0.092 | -0.171 | 0.164 | 0.596 | 0.913 | 0.843 | 0.662 | 1.258 | 0.893 |
| SLC5A2 | AVB | MR Egger | 8 | 0.085 | 0.158 | 0.147 | 0.585 | 1.088 | 1.172 | 0.816 | 1.452 | 0.893 |
| SLC5A2 | LBBB | MR Egger | 8 | 0.150 | 0.279 | 0.148 | 0.351 | 1.161 | 1.322 | 0.869 | 1.552 | 0.893 |
| SLC5A2 | PT | MR Egger | 8 | -0.175 | -0.328 | 0.177 | 0.359 | 0.839 | 0.720 | 0.594 | 1.186 | 0.893 |
| SLC5A2 | RBBB | MR Egger | 8 | -0.231 | -0.432 | 0.148 | 0.170 | 0.794 | 0.649 | 0.594 | 1.061 | 0.893 |
| VEGFA/SLC29A1 | AF | MR Egger | 6 | 0.274 | 0.511 | 0.189 | 0.221 | 1.315 | 1.668 | 0.908 | 1.903 | 0.893 |
| VEGFA/SLC29A1 | AVB | MR Egger | 6 | 0.073 | 0.137 | 0.214 | 0.750 | 1.076 | 1.146 | 0.707 | 1.637 | 0.893 |
| VEGFA/SLC29A1 | LBBB | MR Egger | 6 | 0.319 | 0.596 | 0.281 | 0.321 | 1.376 | 1.815 | 0.792 | 2.388 | 0.893 |
| VEGFA/SLC29A1 | PT | MR Egger | 6 | 0.534 | 0.998 | 0.232 | 0.083 | 1.705 | 2.712 | 1.082 | 2.687 | 0.893 |
| VEGFA/SLC29A1 | RBBB | MR Egger | 6 | 0.072 | 0.135 | 0.173 | 0.698 | 1.075 | 1.145 | 0.766 | 1.509 | 0.893 |
| RXRB | RBBB | MR Egger | 14 | 0.021 | 0.040 | 0.111 | 0.850 | 1.022 | 1.041 | 0.822 | 1.269 | 0.902 |
| ABCC8/KCNJ11 | AF | Weighted median | 3 | -0.031 | -0.058 | 0.103 | 0.761 | 0.969 | 0.943 | 0.793 | 1.185 | 0.904 |
| SLC5A2 | PT | Weighted median | 8 | -0.022 | -0.041 | 0.077 | 0.775 | 0.978 | 0.960 | 0.841 | 1.138 | 0.904 |
| ABCC8/KCNJ11 | AVB | MR Egger | 3 | 0.074 | 0.137 | 0.409 | 0.887 | 1.076 | 1.147 | 0.483 | 2.397 | 0.907 |
| RXRB | PT | MR Egger | 14 | -0.016 | -0.029 | 0.132 | 0.907 | 0.984 | 0.971 | 0.760 | 1.274 | 0.907 |
| RXRB | LBBB | Weighted median | 14 | 0.012 | 0.022 | 0.051 | 0.821 | 1.012 | 1.022 | 0.915 | 1.118 | 0.927 |
| PPARG | LBBB | Inverse variance weighted | 26 | -0.004 | -0.007 | 0.027 | 0.884 | 0.996 | 0.993 | 0.946 | 1.049 | 0.936 |
| ABCC8/KCNJ11 | AVB | Inverse variance weighted | 3 | -0.007 | -0.013 | 0.090 | 0.938 | 0.993 | 0.987 | 0.833 | 1.184 | 0.974 |
| SLC5A1 | LBBB | Inverse variance weighted | 6 | -0.003 | -0.006 | 0.068 | 0.960 | 0.997 | 0.994 | 0.872 | 1.138 | 0.978 |
| GANC | RBBB | Wald ratio | 1 | -0.003 | -0.005 | 0.166 | 0.987 | 0.997 | 0.995 | 0.720 | 1.381 | 0.987 |
| ABCC8/KCNJ11 | AVB | Weighted median | 3 | -0.006 | -0.011 | 0.096 | 0.952 | 0.994 | 0.989 | 0.824 | 1.200 | 0.992 |
| PPARG | AVB | Weighted median | 26 | -0.001 | -0.001 | 0.039 | 0.984 | 0.999 | 0.999 | 0.926 | 1.078 | 0.992 |
| SLC5A1 | AF | Weighted median | 6 | 0.009 | 0.018 | 0.088 | 0.915 | 1.009 | 1.018 | 0.849 | 1.200 | 0.992 |
| SLC5A1 | LBBB | Weighted median | 6 | 0.001 | 0.001 | 0.080 | 0.992 | 1.001 | 1.001 | 0.855 | 1.171 | 0.992 |

| **Table S6. Detailed information of all sensitivity results in two-sample MR analysis** | | | | | | |
| --- | --- | --- | --- | --- | --- | --- |
| **Exposure** | **Outcome** | **Egger_intercept** | **P_pleiotropy** | **Q** | **Q_df** | **P_heterogeneity** |
| ABCC8/KCNJ11 | AF | 0.002 | 0.882 | 1.22 | 2 | 0.543 |
| ABCC8/KCNJ11 | AVB | -0.002 | 0.873 | 0.07 | 2 | 0.965 |
| ABCC8/KCNJ11 | LBBB | -0.012 | 0.375 | 2.48 | 2 | 0.289 |
| ABCC8/KCNJ11 | PT | 0.003 | 0.846 | 1.75 | 2 | 0.416 |
| ABCC8/KCNJ11 | RBBB | 0.009 | 0.677 | 5.08 | 2 | 0.079 |
| GLP1R | AF | -0.011 | 0.614 | 1.12 | 2 | 0.570 |
| GLP1R | AVB | -0.001 | 0.947 | 0.03 | 2 | 0.983 |
| GLP1R | LBBB | -0.007 | 0.731 | 0.25 | 2 | 0.881 |
| GLP1R | PT | 0.014 | 0.561 | 0.76 | 2 | 0.684 |
| GLP1R | RBBB | 0.015 | 0.499 | 1.14 | 2 | 0.565 |
| PPARG | AF | 0.003 | 0.329 | 63.69 | 23 | 0.000 |
| PPARG | AVB | 0.000 | 0.921 | 15.63 | 25 | 0.925 |
| PPARG | LBBB | 0.001 | 0.462 | 17.03 | 25 | 0.881 |
| PPARG | PT | 0.003 | 0.194 | 18.73 | 25 | 0.810 |
| PPARG | RBBB | -0.002 | 0.342 | 30.38 | 25 | 0.210 |
| RXRB | AF | 0.000 | 0.930 | 11.46 | 13 | 0.573 |
| RXRB | AVB | 0.001 | 0.792 | 2.99 | 13 | 0.998 |
| RXRB | LBBB | 0.000 | 0.994 | 22.70 | 13 | 0.045 |
| RXRB | PT | 0.002 | 0.476 | 5.50 | 13 | 0.962 |
| RXRB | RBBB | -0.001 | 0.629 | 4.20 | 13 | 0.989 |
| SLC5A1 | AF | -0.003 | 0.631 | 0.40 | 5 | 0.995 |
| SLC5A1 | AVB | -0.002 | 0.749 | 2.78 | 5 | 0.733 |
| SLC5A1 | LBBB | -0.005 | 0.358 | 1.39 | 5 | 0.925 |
| SLC5A1 | PT | -0.006 | 0.325 | 2.87 | 5 | 0.720 |
| SLC5A1 | RBBB | -0.006 | 0.446 | 9.13 | 5 | 0.104 |
| SLC5A2 | AF | 0.003 | 0.378 | 7.06 | 7 | 0.422 |
| SLC5A2 | AVB | -0.002 | 0.465 | 1.51 | 7 | 0.982 |
| SLC5A2 | LBBB | 0.000 | 0.912 | 4.20 | 7 | 0.756 |
| SLC5A2 | PT | 0.003 | 0.390 | 4.19 | 7 | 0.757 |
| SLC5A2 | RBBB | 0.001 | 0.647 | 4.24 | 7 | 0.751 |
| VEGFA/SLC29A1 | AF | -0.003 | 0.502 | 3.73 | 5 | 0.588 |
| VEGFA/SLC29A1 | AVB | 0.000 | 0.914 | 6.25 | 5 | 0.283 |
| VEGFA/SLC29A1 | LBBB | -0.004 | 0.500 | 12.07 | 5 | 0.034 |
| VEGFA/SLC29A1 | PT | -0.007 | 0.184 | 8.34 | 5 | 0.138 |
| VEGFA/SLC29A1 | RBBB | -0.001 | 0.858 | 3.99 | 5 | 0.551 |
